# A cell-based computational model of early embryogenesis coupling mechanical behaviour and gene regulation

**DOI:** 10.1038/ncomms13929

**Published:** 2017-01-23

**Authors:** Julien Delile, Matthieu Herrmann, Nadine Peyriéras, René Doursat

**Affiliations:** 1 BioEmergences Laboratory (USR3695), CNRS, Université Paris-Saclay, Gif-sur-Yvette 91198, France; 2 Complex Systems Institute Paris Ile-de-France (ISC-PIF, UPS3611), CNRS, Paris 75013, France

## Abstract

The study of multicellular development is grounded in two complementary domains: cell biomechanics, which examines how physical forces shape the embryo, and genetic regulation and molecular signalling, which concern how cells determine their states and behaviours. Integrating both sides into a unified framework is crucial to fully understand the self-organized dynamics of morphogenesis. Here we introduce MecaGen, an integrative modelling platform enabling the hypothesis-driven simulation of these dual processes via the coupling between mechanical and chemical variables. Our approach relies upon a minimal ‘cell behaviour ontology' comprising mesenchymal and epithelial cells and their associated behaviours. MecaGen enables the specification and control of complex collective movements in 3D space through a biologically relevant gene regulatory network and parameter space exploration. Three case studies investigating pattern formation, epithelial differentiation and tissue tectonics in zebrafish early embryogenesis, the latter with quantitative comparison to live imaging data, demonstrate the validity and usefulness of our framework.

## Abstract (web summary)

Embryonic development is a complex process where genetic and biochemical information direct morphogenesis. Here the authors describe MecaGen, an agent-based model and simulation platform of multicellular development designed to allow a quantitative comparison between simulations and real biological data.

Understanding how multicellular organisms composed of complex structures are able to develop from a single cell, the fertilized egg, raises fundamental questions both of a biomechanical nature and of a genetic and biochemical nature. Metaphorically, one could say that an embryo ‘sculpts itself' at the same time that it grows and ‘paints itself'^1^, where colours represent differentiated cell types. Shapes and gene expression patterns emerge concurrently from the self-organization of a myriad of cells, affecting each other in a feedback loop. We present an integrated computational model and simulation platform of these dual processes, called MecaGen.

On the one hand, cell biomechanics investigates how physical forces and deformations exerted and sustained by cells progressively transform the embryo, defining morphogenesis^2^. Spatial cell behaviours, such as shape change, migration or oriented division, are controlled by molecular mechanisms and continuous remodelling of the cytoskeleton in interaction with the plasma membrane and cell junctions. Several theoretical principles have been put forward to explain these phenomena. Extending Holtfreter's concept of ‘tissue affinity'^3^, the differential adhesion hypothesis (DAH) states that cells favour contacts with neighbours of higher binding strength, hence minimize the adhesive free energy of the tissue^4^. Later refinements to DAH identified cortical tension as a key factor in the interfacial surface tension^5^,^6^. The cellular Potts model, a multivalued Ising lattice where each cell covers a patch of pixels, formalized these ideas in a Monte Carlo framework and applied them to cell sorting experiments^7^,^8^. It was also augmented with chemotaxis, cell division and cell death, by modifying the Hamiltonian function^9^. Alternative implementations of DAH were derived from vertex-based models in which vertices represent intersections between cell junctions^10^,^11^. Other 3D mechanical models of multicellular systems, including ours, follow an agent- and force-based approach using one particle or several ‘subcellular elements', ellipsoidal or polyhedral, per cell^12^,^13^.

On the other hand, the genetic and signalling aspect of developmental biology can be summarized by the formal concept of gene regulatory network (GRN), which shifts the focus from single genes to molecular interactions among multiple genes and signalling pathways^14^. The topology of a GRN is determined by certain DNA sequences called cis-regulatory modules, in which transcription factors bind to appropriate sites and form molecular complexes, triggering or hindering the recruitment of RNA-polymerase (RNAP) to initiate or block gene transcription. Various computational GRN models were proposed^15^, such as binary networks with Boolean cis-regulatory modules functions^16^, or sets of kinetic differential equations evaluating the probability that RNAP will bind to a promoter sequence^17^,^18^.

Yet, embryonic development cannot be described solely from either the mechanical or the molecular and genetic perspectives. The key to understanding morphogenesis lies in their tight mutual coupling and interplay, constitutive of the cell biology level. On the one hand, we need to take into account the causal links^19^ from signals to forces through the modulation of the cytoskeleton and surface adhesion dynamics downstream of the GRN activity. On the other hand, the model also needs to include the influence of forces on signals through the deformation and mechanical stress of the cellular tissue due to local cell rearrangements, which modify the signalling environment via ligand-gated transduction and mechanotransduction.

While there is a great number of specific models of pattern formation or shape generation tailored to given species and particular embryonic events, only a handful of software tools propose a generic combination of mechanical and chemical rules in the global context of multicellular development (Supplementary Table 1 and Supplementary References). None of these systems, however, offers a fully integrated architecture relating molecular signalling, genetic regulation and structural changes to one another: Ingeneue^20^ embeds gene networks in sheets of cells, but does not distinguish heterogeneous cell types or behaviours; VirtualLeaf^21^ and Cellzilla^22^ both simulate the transport of chemical signals across a polygonal mesh of elastic compartments, the latter also allowing the specification of complex chemical networks, but only for plant growth in 2D and without coupling between GRN and biomechanics; CompuCell3D (ref. 23), an on-lattice voxel-based model without freely moving components, provides a simulation environment merging the cellular Potts model with chemical fields and diffusion equations. Macroscopic cell behaviours are linked to intracellular molecular concentrations simulated using an external Systems Biology Markup Language (SMBL) library such as libRoadRunner; CellSys^24^ focuses on multi-agent simulations of tumour growth and LBIBCell^25^ couples viscoelastic cell mechanics with reaction-advection-diffusion solvers, but neither includes GRN specification.

Our open-source modelling platform, MecaGen, links GRN dynamics to the control of individual cell behaviours in order to account for a wide range of morphogenetic events constitutive of animal early embryogenesis. Aiming for a trade-off between computational feasibility and physical realism, it relies on an agent-based model with one particle per cell obeying a set of ordinary and partial differential equations, both Newtonian dynamics and reaction kinetics. Following an ‘emergent modelling' approach—the hallmark of all complex systems including morphogenesis—the main originality of MecaGen resides in the adaptation and combination of a number of relatively simple laws at the cellular level into an integrated framework able to account for qualitatively varied collective phenomena. Similar to the successful models of bird flocks and human crowds^26^, which summarize collective motion effects in a few ‘separation' and ‘alignment' forces at the level of each individual without including their neural activity or sensorimotor skills, our model is set at the level of the multicellular system and reproduces embryonic episodes from a few mechanical and genetic principles at the level of each cell without detailing the molecular structure of their cytoskeleton or DNA. In that sense, and like the other frameworks mentioned above, our elementary rules are not a lot more sophisticated than the classical cellular automata (Turing patterns)^27^ and generative grammars (L-systems)^28^ of pattern formation (stripes, spots, branches) in animals and plants—yet at the same time they are capable of exhibiting complex developmental structures that none of these models can. The schematic representations of biological objects in MecaGen are also designed to allow comparisons between the simulated specimen and a ‘reconstructed specimen' obtained by algorithmic processing of 3D+time microscopy imaging^29^,^30^,^31^,^32^. In sum, this framework should be a valuable tool for developmental biologists to create a model of the spatiotemporal transformations of embryonic tissues and calculate their quantitative difference with biological data. In addition to two examples based on artificial data, we demonstrate how MecaGen models the early steps of zebrafish embryogenesis and epiboly movements, together with an exploration of parameter space and comparison to imaging data. These case studies highlight MecaGen's ability to formalize some of the major processes underlying early embryonic development.

## Results

### The MecaGen model

**Figure 1:**
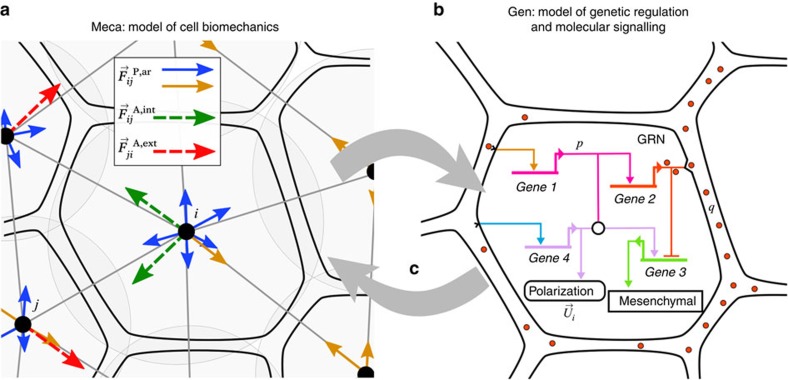
Schematic overview of the MecaGen model coupling the cell's biomechanical properties to its biochemical activity. Mechanical parameters are specified by the gene expression dynamics and molecular state. Conversely, spatial rearrangements among cells impact protein synthesis via signalling and mechanical stress. (**a**) Meca: cell shapes are idealized as ellipsoids (pale grey) represented by a centre (black dot) and two radii (Supplementary Fig. 1). Edges connecting centres materialize cell neighbourhoods, derived from metric and topological criteria. Neighbouring cells exert ‘passive' and ‘active' forces on one another (Supplementary Note 2, equation (3)). Passive relaxation forces (solid arrows), in particular attraction-repulsion 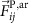, maintain volume integrity via adhesion and cortical tension coefficients (equation (14) and Supplementary Figs 3–5). Attractive forces (orange) point towards the neighbours, while repulsive forces (blue) point away from them. Active behavioural forces (dashed arrows), exerted at the level of protrusions or apical constriction and involved in cell intercalation, comprise pairs of ‘intrinsic' components 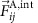 (green) and ‘extrinsic' components 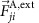 (red; equation (21) and Fig. 5e). During protrusion, intrinsic forces result from the cell's cortical cytoskeleton maintaining its shape, while extrinsic forces result from the traction on neighbouring cells through protrusive activity (here, to the left). (**b**) Gen: the biochemical model relies on a gene regulatory network (GRN), associated with concentration variables of intracellular proteins and extracellular ligands, driven by chemical kinetics (synthesis, secretion, binding) and reaction-diffusion equations (30)–(47). (**c**) MecaGen: both sides are coupled via a cell behaviour ontology of three cell ‘archetypes': epithelial, mesenchymal and idle (Fig. 2) corresponding to mutual relationships between Meca and Gen variables (equations (48)–(58)).

The model comprises two parts, ‘Meca' and ‘Gen', and their coupling. The Meca side (Fig. 1a) consists of a discrete-element method applied to ellipsoidal cells that obey an overdamped equation of motion of the type 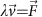, where 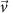 is the cell's velocity and *λ* is a viscosity coefficient—a common abstraction of the motion dynamics in a complex and densely packed molecular environment. Similar to a low Reynolds number in fluid mechanics, inertial forces 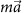 are negligible here because cells are very small entities with ambivalent solid-fluid biophysical properties^33^ that are submitted to strong ‘sticky' interactions. Forces 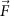 are calculated on each cell *i* by summing 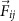 components over a topological neighbourhood *N*_*i*_ containing the cells *j* in contact with it, based on a variant of the Voronoi diagram (a purely metric neighbourhood, based on distances, is not viable as it leads to collapsing volumes when adhesion strength is high). Two types of forces are modelled: (1) ‘passive' relaxation forces representing cell-cell adhesion, which include attraction-repulsion components 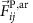 derived from an elastic-like interaction potential with adhesion coefficients *w*, as well as planarity conservation components 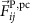 expressed in epithelial domains; and (2) ‘active' behavioural forces 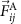 representing a schematic view of polarization, mesenchymal cell protrusion and epithelial cell-junction remodelling, which cause cells to intercalate themselves between neighbours, migrate and generally move the system away from equilibrium (Supplementary Notes 1 and 2, equations (1)–(29)). The essential distinction between passive and active forces is that the former are always felt and exerted among all cells at any time, while the latter are present only when certain behaviours, such as protrusion, are genetically expressed via differentiation into specific types (see below). Here cell behaviours are not modelled through explicit geometric deformations of cell shapes, but through mechanical interactions. In particular, mesenchymal protrusion does not result from varying directly the resting lengths of the ellipsoid axes, but from introducing pairs of forces between a protruding cell and its neighbours.

The Gen side of the model (Fig. 1b) deals with chemical signalling and GRNs. It is composed of three types of rules abstracting the cascade of signalling reactions and metabolic activity: (1) intracellular ordinary differential equations d**p**/d*t*=*f*(**p**, **g**, **q**, ***ρ***) describing the transcriptional GRN dynamics, where **p** represents a list of intracellular protein concentrations, **g** gene expression levels, **q** extracellular ligands, ***ρ*** membrane receptors and *f* is a Boolean function containing a logical combination of cis-regulatory promoters and repressors of gene expression; (2) extracellular reactions, transport and diffusion of ligands, written as partial differential equations involving ∂**q**/∂*t* and flux vectors 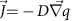 (Supplementary Fig. 10); and (3) protein secretion and signal transduction, which link the internal protein concentrations **p** to the spatialized ligand concentrations **q** via ligand-receptor binding on the cell membrane (Supplementary Note 3, equations (30)–(47)).

**Figure 2:**
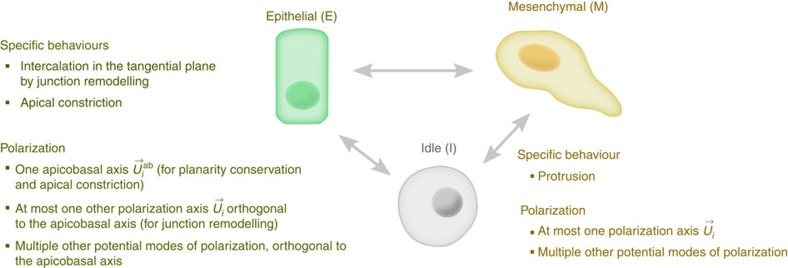
Cell behaviour ontology (CBO) foundations of the MecaGen coupling between mechanical and molecular states. Cells can transition between three ‘archetypes': mesenchymal (M), epithelial (E) and the default, idle (I), controlled by the GRN topology and dynamics. Protrusion, most apparent in M cells, is a treadmilling activity based on adhesion (similar to tracked vehicles), which is regulated to avoid sliding at the contact surface area, and can induce an intercalation motion between neighbour cells (Supplementary Figs 7 and 8). It rests on a polarization axis 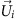, generally created by an asymmetrical distribution of external ligands and internal substances (Fig. 5c). Like mesenchymal cells, epithelial cells are able to intercalate themselves between other E cells, except that they remain in the tangential plane of the epithelium. This is due to a property of ‘planarity conservation' supported by another type of passive relaxation forces 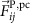 (Supplementary Note 2, equation (17)) and an additional apicobasal polarization axis 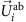 (Fig. 4c). They can also exhibit active apical constriction (similar to purse strings), but this behaviour is not implemented in the current version of the model. Differentiation into E requires signalling by other surrounding epithelial cells to create and maintain apicobasal polarity (equation (9) and Supplementary Fig. 2), otherwise an isolated cell reverts to I. Finally, both M and E cells can be polarized by multiple ‘potential' mechanisms throughout development: chemotaxis along concentration gradients, propagation of alignment via cell contacts, polar induction from nearby protruding cells, and randomized orientation by blebbing (equations (25), (52)–(54)).

Finally, Meca and Gen are coupled via relationships between mechanical and chemical variables (Fig. 1c). The present version includes the control of attraction forces by surface densities of adhesion molecules, the control of differentiation and specific behaviour by gene regulation, the control of polarization axes by ligands and neighbouring cells, and the control of gene regulation by mechanotransduction (Supplementary Note 4, equations (48)–(58)). MecaGen coupling consists of identifying a relevant entry in a cell behaviour ontology (CBO) from a ‘cell state' depending on the set of intracellular protein concentrations (Fig. 2). Here, cells are classified into the three generic ‘archetypes' observed during the early stages of embryogenesis across a wide variety of animal embryos: mesenchymal cells (M), epithelial cells (E) and ‘idle' cells (I). The latter exhibit no active biomechanical behaviour but still offer resistance to deformation. Other cell archetypes appearing at later developmental stages through further differentiation are not specified here. Extraembryonic structures, such as the yolk and enveloping layer in the zebrafish, are handled by adding customized code to the MecaGen platform to model particles and forces similar to cells but not belonging to an archetype.

Like gene expression, differentiation into M or E is controlled by two specific ‘output' nodes of the GRN representing Boolean functions of proteins *f*(**p**) (Supplementary Note 4, ‘Control of Archetype'), with a limited number of inputs under the simplified representation of molecular dynamics adopted here. Then, through the CBO, each archetype is associated with specific active biomechanical behaviours that lead to a deformation of the cell assembly. Mesenchymal cells are motile entities displaying monopolar or bipolar protrusive activity along a polarization axis 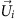, while epithelial cells possess an extra apicobasal polarization axis 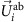 which contributes to the intercalating behaviour and also causes the formation of compartments in the embryo via the planarity conservation forces. Conversely, the aggregate effect of the cells' mechanical activity creates a spatial reorganization of the tissue that feeds back onto the GRN dynamics through a modulation of cell-cell contacts, hence signalling, and through mechanotransduction.

### Simulation of typical morphogenetic processes

The features of MecaGen are illustrated here by three case studies, which summarize major types of developmental events underlying animal early embryogenesis, and showcase the versatility and usefulness of the MecaGen model: (i) tissue patterning, where cells interpret positional clues to establish domains of distinct fate; (ii) differentiation of idle cells into epithelial cells, leading to the establishment of borders and formation of compartments; and (iii) tissue tectonics, more specifically the first phase of epiboly in zebrafish early embryogenesis resulting from the collective behaviour of thousands of cells^34^. In all three cases, the morphogenetic phenomenon depends on the specific architecture and dynamics of the GRN.

### Tissue patterning controlled by a GRN toggle-switch circuit

**Figure 3:**
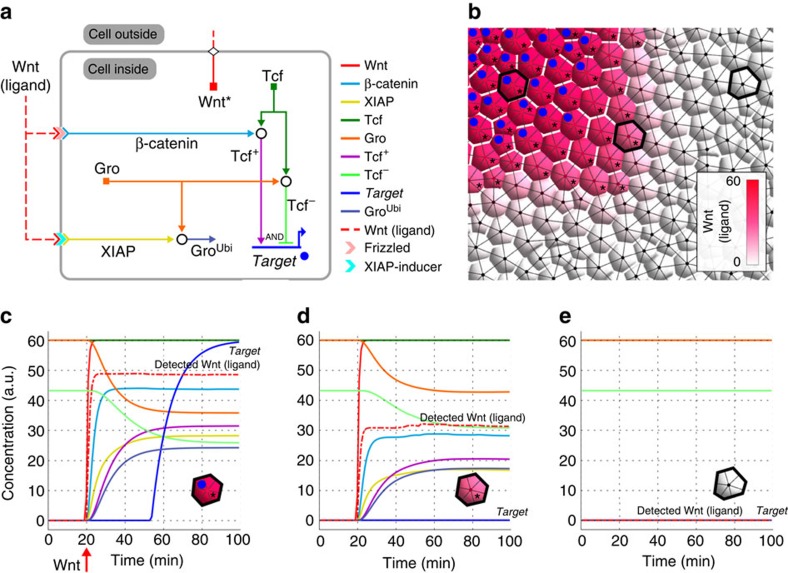
Example of genetic regulation and molecular signalling. This example based on *Drosophila* shows the possibility of (neural) cell fate specification and pattern formation in a spatially explicit simulation of tissue (Supplementary Movie 1). Cells divide sporadically, increasing their number from 880 to 1,125 (Supplementary Note 2, equations (24)–(29)). (**a**) The GRN contains a signal-mediated toggle switch using a single *Target* gene, upregulated (resp. downregulated) by a Tcf+ (resp. Tcf−) protein complex (equations (30)–(32)). This complex results from the reaction (black circle) of Tcf with an intracellular cofactor, β-catenin (resp. Groucho, or Gro; equations (33)–(35)). The internal release of β-catenin (resp. XIAP) is triggered by ligand-receptor binding (chevrons) and transduction (equation (40)). In turn, XIAP induces the ubiquitination of Gro and its degradation (equation (36)). (**b**) Partial snapshot at *t*=95 min (showing about 120 cells). Production of Wnt protein was turned on at *t*=20 min in one region (asterisks; equation (38)), provoking the secretion of Wnt ligand (equation (39)) and its extracellular diffusion (pink gradient; equations (43)–(46)). At *t*>50 min, the expression of *Target* shoots up in cells that have received enough Wnt ligand (blue dots). (**c**–**e**) Temporal evolution of protein concentrations in different regions. Initially, Gro, Tcf, Tcf− and receptors are ubiquitously present. (**c**) Cells bathing in high levels of Wnt ligand express *Target*. (**d**) Cells bordering the source region receive less Wnt ligand, thus *Target* remains silent. (**e**) Cells far from the Wnt sources display no activity. See parameters in Supplementary Table 2.

Morphogenesis in the animal embryo rests on the diversification of cell fates towards the creation of various structures. Heterogeneous cues cause the individuation and appearance of distinct morphogenetic fields, which later become compartments. The ability of MecaGen to account for the emergence of differentiation as a consequence of genetic regulation and molecular signalling is demonstrated by embedding a GRN subcircuit, the signal-mediated toggle switch^14^, into a spatially explicit simulation of tissue composed of hundreds of cells (Fig. 3 and Supplementary Movie 1). This toggle switch highlights a generic developmental mechanism, one that produces two distinct cell fates depending on the expression or silence of some *Target* gene, under the control of an activation/repression switch involving a transcription factor (Tcf) downstream of a signalling pathway. A similar GRN motif underlies, for example, the specification of cell fate via Notch signalling in the peripheral nervous system development of *Drosophila*^35^.

In the present case study, we illustrate the toggle switch mechanism through the operation of the Wnt signalling pathway in *Drosophila* cells^36^. Following a simplified scenario, Wnt ligand-gated transduction leads to the production of both β-catenin and Groucho (Gro) proteins, which interact with Tcf to form two complexes, Tcf+ and Tcf− (Fig. 3a). These play the role of cis-regulatory inputs that concurrently activate and repress *Target*, ultimately determining the cell's fate. Initially, assuming a constant level of Tcf in all cells, the system is stimulated by turning on the internal production and secretion rates of Wnt in a subset of the cells (Fig. 3b, asterisks), then letting Wnt diffuse externally and create a concentration gradient across the tissue (red levels). After a while, the cells that have been reached by a sufficient level of extracellular Wnt ligand start expressing *Target* (blue dots and Fig. 3c). The other cells, whether they are inside or outside of the region that produces intracellular Wnt proteins (Fig. 3d,e), do not differentiate into the *Target* type.

In sum, due to the Boolean logic of the toggle-switch GRN subcircuit, tissue patterning into different cell states and their corresponding fates is determined by the specific activation level of a signalling pathway, through secretion, diffusion, transduction and reactions with internal transcription factors. Other generic GRN subcircuits such as the double negative gate motif^19^ can also be efficiently implemented in MecaGen. As the next two case studies also show, our platform offers an integrative modelling and simulation framework in the 3D space of a tissue or the entire developing embryo, able to relate extracellular ligand concentrations to intracellular cell states via signalling pathways.

### Compartment formation by induction and epithelialization

**Figure 4:**
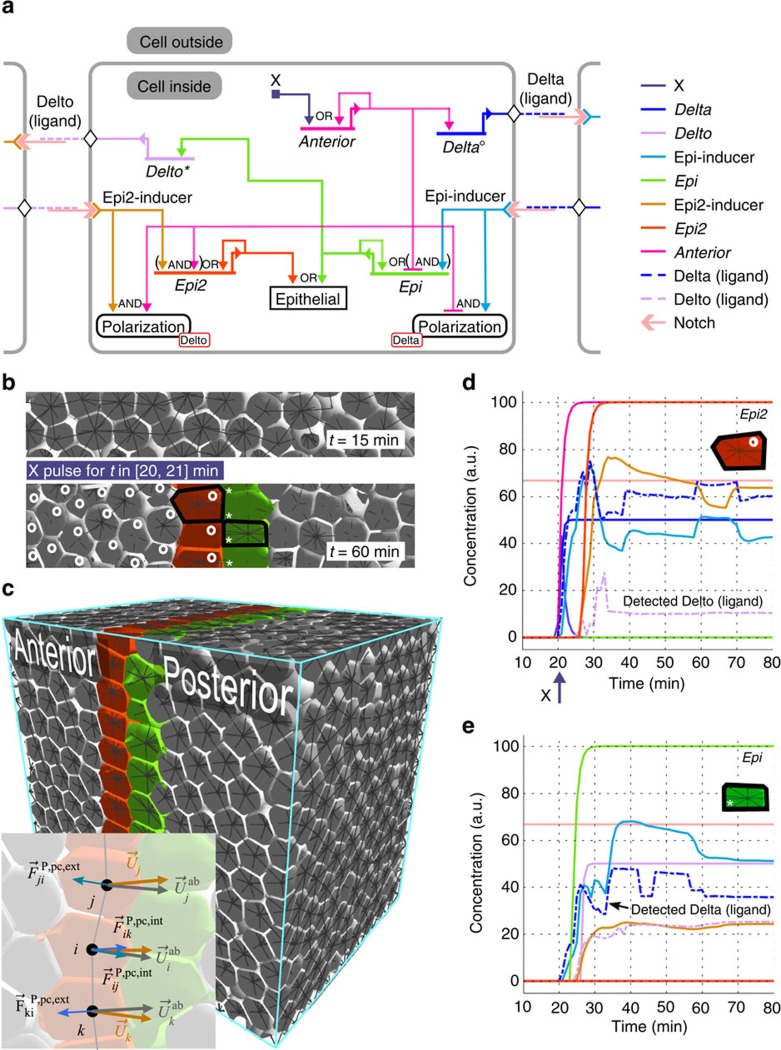
Example of boundary formation and epithelialization. See Supplementary Movie 2. (**a**) The GRN implements a Delta-Notch mechanism of mutual induction of the type found in the *Drosophila* wing. Right half: Intracellular protein X stimulates Anterior, a self-activating transcription factor (left cells in **b**,**c**). Anterior promotes the synthesis of a transmembrane ligand Delta (circles), which binds to a Notch receptor on the nearest cells. This produces Epi-inducer by transduction (Supplementary Note 3, equation (41)) and, in the absence of Anterior, leads to the synthesis of Epi. The consequence is that only cells in contact with, but outside of the X domain express *Epi* (green cells) and adopt a polarized epithelial type oriented along the gradient of Delta. Left half: Symmetrically, the Epi cells synthesize another ligand, Delto (asterisks), which leads to the epithelialization of Anterior cells in contact with them, via Epi2 (orange cells). (**b**) Zoom on a 2D slice of tissue. After applying a pulse of X on the left, two adjacent rows of cells have differentiated into Epi2 and Epi at the boundary. (**c**) View of the 1,683-cell cubic domain at a late stage (neighbourhood edges partially visible). Cells do not divide here. Inset: Apicobasal polarization axes (grey) and partial planarity conservation forces (blue) on three Epi2 cells (Supplementary Fig. 6). (**d**,**e**) Temporal evolution of protein concentrations in and around one boundary cell. The irregular profiles of certain curves are caused by spatial rearrangements and consequent fluctuations in transduction signals and messengers, as E cells elongate and align in a planar way. See parameters in Supplementary Table 3.

After different domains of genetic expression have started forming in an embryonic tissue, separation of cell fates is generally complemented with a restriction of the cell lineage to compartments. It requires identifying the population of cells lying between two compartments and, often, mutual induction of these border cells towards a sharpening of the boundary between them^37^. This is exemplified by the Delta-Notch signalling mechanism, found in *Drosophila*^38^ and largely conserved across evolution, which is implemented here. Unlike continuum or fixed-lattice models of gradients, MecaGen simulations also include mechanical cell motion and reshaping near the boundary. This case study illustrates how MecaGen can support the control and emergence of a spatial pattern of epithelial differentiation, coupled with slight movements in the vicinity of the boundary, as newly polarized cells start aligning on a plane (Fig. 4 and Supplementary Movie 2).

Initially, the anterior half of a cubic and homogeneous domain of about 12^3^ idle cells (left side of Fig. 4b,c) is exposed to a pulse of protein signal X for a short period of time. According to the GRN in each cell (Fig. 4a), X promotes the expression of an autoregulated gene, *Anterior*, whose product interacts with transduction paths from Notch receptors to give rise to two vertical monolayers of epithelial cells, Epi and Epi2, displaying a sharp boundary surface at their junction. This process follows an anterior→posterior→anterior causal chain of events: (1) the Anterior protein activates the synthesis and secretion (surface expression) of a ligand Delta in the whole anterior domain exposed to X (Fig. 4b, circles); (2) the Delta-Notch transduction induces Epi but only outside of the anterior domain (Anterior is a repressor of Epi), hence only in the posterior border cells (green cells); (3) in turn, these cells synthesize and release another ligand, Delto (asterisks), whose transduction induces the expression of Epi2 only in the anterior domain (Anterior is a promoter of Epi2), hence only in the anterior border cells (orange cells). The temporal evolution of intracellular protein and extracellular ligand concentrations in border cells are shown in Fig. 4d,e. This generic segmentation mechanism has been described, for example, at the dorsoventral border of the developing *Drosophila* wing^39^ or in the somitogenesis of the chicken^40^,^41^, with Fringe and Serrate playing the roles of Anterior and Delto.

Downstream of the transcriptional regulation (Fig. 4a, output nodes), activation of the epithelial archetype E is conditioned by the presence of a sufficient level of Epi (resp. Epi2) protein in the posterior (resp. anterior) cells, which also adopt a polarization axis 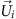 oriented towards the higher concentration of Delta (resp. Delto) facing them (Fig. 4c and Supplementary Note 4, ‘Chemotactic mode', equation (52)). Additionally, in order to acquire a fully polarized state, epithelial cells must be subjected to lateral reinforcement of their internal polarization from at least two similar neighbouring epithelial cells. This prevents a single E cell from exhibiting a specific epithelial mechanical behaviour if it is surrounded by non-E cells. Finally, once an epithelial cell is fully differentiated, including its apicobasal polarity 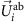 calculated geometrically from the neighbourhood edges (Supplementary Note 2, equation (9)), it starts generating planarity conservation forces 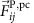, which tend to align neighbouring E cells laterally (Fig. 4c and Supplementary Note 2, equations (15)–(17)), hence form and maintain a sharp compartment boundary.

### Embryonic patterning emerging from cell polarization cues

**Figure 5:**
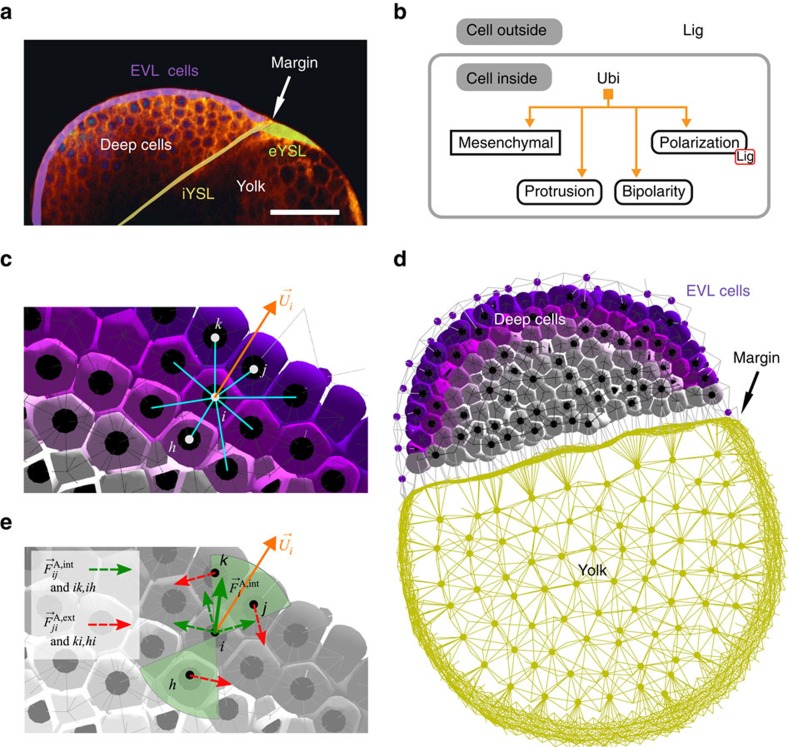
Example of collective behaviour during the zebrafish epiboly. See Supplementary Movie 3. (**a**) 2D section from live imaging 3D data (‘oblong' stage, 3.7 hpf), highlighting the EVL, the external and internal YSL, and the interface between the blastoderm and the yolk cell (credit: BioEmergences). Scale bar 150 μm. (**b**) Simplified GRN controlling the bipolar protrusion of mesenchymal deep cells via protein Ubi, oriented by a gradient of extracellular ligand Lig. (**c**) Their polarization axes 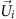 are oriented by chemotaxis (Supplementary Note 3, equations (43)–(46)) along a radial gradient of ligand (purple) released from the EVL (not shown). (**d**) Sagittal section of the whole simulated embryo (4 hpf), containing 1,595 deep cells (purple and grey polyhedra) and showing the EVL cell centres (purple dots), yolk particles (yellow dots) and yolk membrane (peripheral yellow edges). EVL and yolk take part only in passive relaxation forces. (**e**) In the bipolar domain of cell *i* (green cones) containing three neighbours, protrusive forces comprise ‘intrinsic' (dashed green arrows) and ‘extrinsic' components (dashed red; equation (21) and Supplementary Fig. 9), resulting in 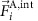 (solid green arrow).

**Figure 6:**
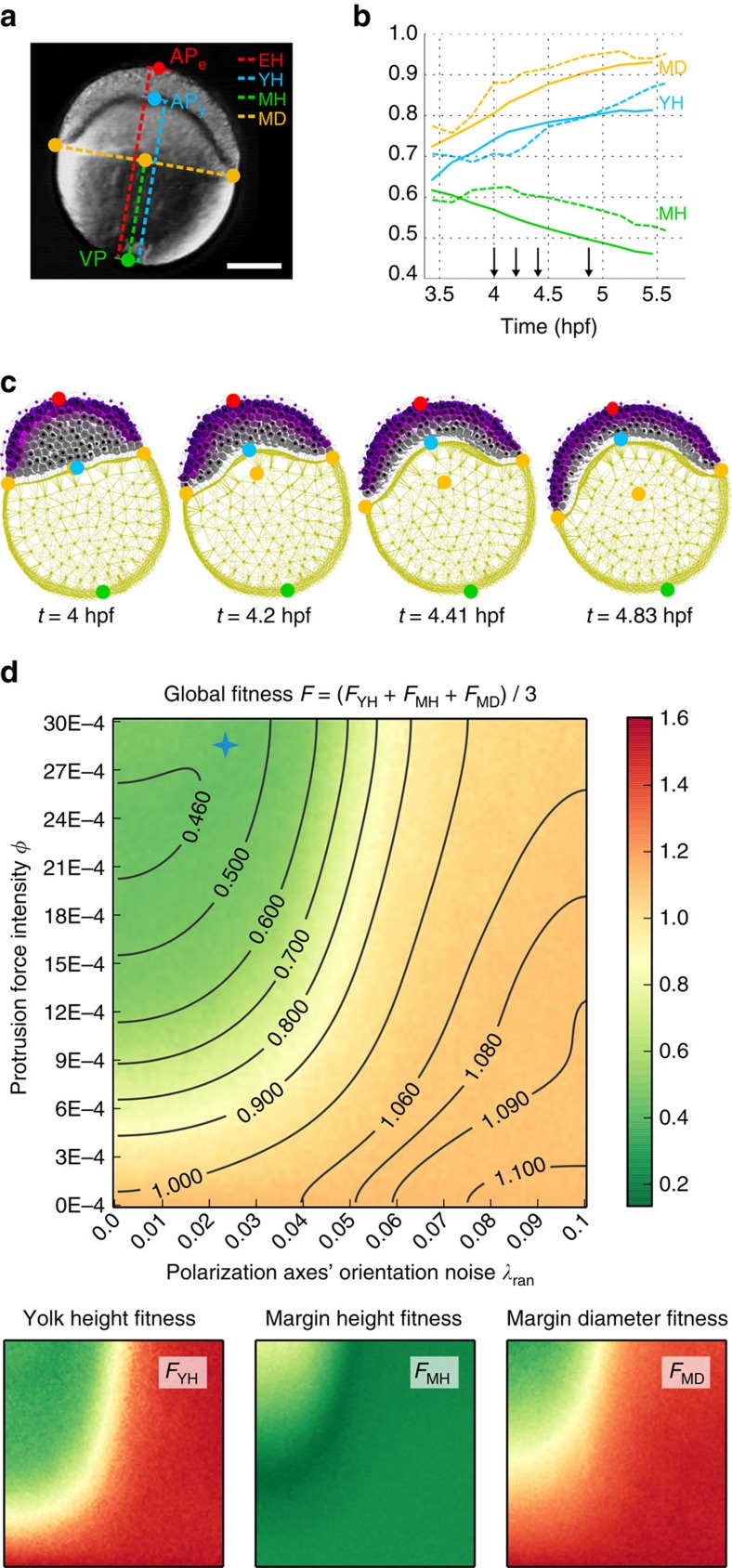
Parameter exploration of the zebrafish epiboly study. See Fig. 5. (**a**) Macroscopic measurements of the epibolic deformation (pasted on a Nomarski 2D picture at the level of the sagittal plane by Karlstrom and Kane^45^, with permission). Landmarks: vegetal pole (VP), animal pole (APe), yolk animal pole (APy). Distances: embryo height (EH), yolk height (YH), margin height (MH) and margin diameter (MD). Scale bar 200 μm. (**b**) Temporal evolution of the last three measurements normalized by EH in the live embryo (dashed lines) and the best simulated embryo (solid lines). (**c**) Snapshots at four intermediate stages (time arrows in **b**). As deep cells divide (mitosis equations (24)–(29) based on empirical data^29^,^32^), their number increases to 3,095. See parameters in Supplementary Table 4. (**d**) Fitness landscapes as a function of the protrusive force intensity *φ* and a noise factor *λ*_ran_ controlling the regularity of the polarization axes' orientation. The global fitness function *F* is the average of the yolk height fitness *F*_YH_, the margin height fitness *F*_MH_ and the margin diameter fitness *F*_MD_. A lower fitness value (green) means a better similarity with the live embryo. The blue cross highlights the parameter values used in **b**, which are: 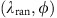 ≈ (0.025, 28.5E-04).

The collective cell rearrangements giving rise to massive tissue deformation is another pervasive feature of early embryogenesis^2^,^42^. To demonstrate how MecaGen allows the study of molecular cues that can both direct the specification of motile behaviour and coordinate the displacements of thousands of individual cells, we model here the first phase of epiboly in the zebrafish embryo, an episode occurring between 3.7 h post-fertilization (hpf) (oblong stage) and 5.3 hpf (50%-epiboly stage)^43^. It is characterized by a flattening of the deep cell mass and its spreading over the yolk towards the vegetal pole (Figs 5 and 6, and Supplementary Movie 3). This mass lies on top of the yolk, between the yolk syncytial layer (YSL) and the enveloping layer (EVL), a dome-shaped population of newly differentiated epithelial cells, forming a circular interface with the YSL called the blastoderm margin (Fig. 5a). Deep cells divide and start to intercalate radially while the yolk bulges inside the blastoderm until it is uniform in depth at all latitudes^44^. We show that polarized protrusive behaviours from the deep cells (of archetype M) are sufficient to account for most of the embryo deformations at these stages.

Mimicking the coupled action of adhesion and actin treadmilling at its tip (Supplementary Figs 7 and 8), cell protrusion is modelled by a pair of equal and opposite forces contributing to the motion of both the acting cell (‘intrinsic' forces 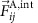) and the neighbours against which it is pushing (‘extrinsic' forces 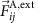; Fig. 5e). A protruding cell only acts upon neighbour cells that belong to the polar domain defined around its polarization axis 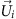 (green cones). Although the fact that signalling molecules could be responsible for cell protrusion orientation during zebrafish epiboly remains to be established^45^, we postulate here that 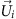 follows a ‘chemotactic' mode of determination based on a radial molecular gradient secreted inward from the EVL (Fig. 5c). Other polarization modes, such as ‘propagation', ‘protrusion-induced' and ‘blebbing', are also available in MecaGen (Supplementary Note 4, equations (52)–(54) and Supplementary Note 2, equation (25)).

To investigate the effect of protrusion forces and their orientation on the dynamics of zebrafish epiboly, we conducted a parameter space exploration along two dimensions (Fig. 6d): the intensity of the protrusive forces, via a coefficient *φ* linking them to adhesion (Supplementary Note 4, equation (57) and Supplementary Note 2, equation (21)), and a polarization noise 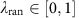 such that the effective vector is 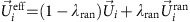, where 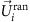 is uniformly drawn in each cell *i* every 7.5 min of simulation time. Therefore, the polarization axes varied from regularly oriented and orthogonal to the EVL surface to completely random, via a linear combination of these two extremes.

The morphologies created by computational simulation were compared to the ones derived from *in toto* embryo imaging by Nomarski video microscopy^45^. This was based on the geometric relationships between five landmarks of the embryo: the vegetal pole (VP), animal pole (APe), yolk animal pole (APy), and the projected left and right margin positions (Fig. 6a,c). The first three landmarks were positioned on the particles of the yolk membrane and EVL that realized a maximally positive or negative dot product with the animal-vegetal axis vector. As epiboly unfolds, four distances are calculated between those points: the embryo height (EH), yolk height (YH), margin height (*MH*) and margin diameter (MD). The last three are divided by the first to obtain normalized metrics, and their evolution is displayed in Fig. 6b. The concurrent rise of YH and MD indicates a transition from the oblong shape to a near spherical shape (‘germ ring' stage, 5.7 hpf), while the decrease in MH corresponds to the progression of the margin towards the vegetal pole.

Using these dynamical measures, we define three fitness functions as the absolute differences between the real and simulated curves: 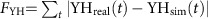, same with *F*_MH_ and *F*_MD_, and a global fitness value *F* as their average (Fig. 6d). Thus the lower these values, the better the simulation. The 2D fitness landscapes obtained by varying *λ*_ran_ and *φ* show that these parameters have counterbalancing effects: a higher protrusive force coupled with a higher polarization noise produce a fitness value similar to a lower force coupled with a lower noise, as indicated by the isoclines of Fig. 6d. The profile of the isoclines also reveals a supralinear relationship between these parameters, as an increase of Δ*λ*_ran_ requires a relative increase of 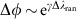 to be counterbalanced (in other words, noise in polarity cues is compensated by stronger active adhesion).

For 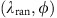 pairs located below the isocline passing through coordinates (9E-04, 0), no macroscopic epiboly behaviour is observed. In addition, we can relate this area to various abnormal microscopic behaviours: for low values of *φ* (that is, low protrusive adhesion forces) the lack of epibolic deformation is caused by the lack of intercalating behaviour at the cellular level; on the contrary, for high values of *φ* and *λ*_ran_ (that is, high protrusive adhesion forces and very noisy radial polarity gradient) cells intercalate inefficiently, slipping on each other like a fluid, and tissue cohesion is lost. This is due to an unequal balance between passive relaxation and active protrusive forces in favour of the latter. In the region above the same isocline, another trade-off exists in the top region, where a higher *φ* induces a better embryo shape (low MD fitness) at the expense of the margin location (high MH fitness). This is due to an abruption of the deep cells from the yolk surface when protrusive forces are too strong.

Overall, we can find sets of parameters leading to a good match between the live and simulated specimen, which shows that our hypothesis (about the oriented protrusion of deep cells being the main drive) is indeed sufficient to quantitatively reproduce the embryo's macroscopic deformation during the first phase of epiboly. It should also be noted that similar emergent behaviours were observed in simulation from either bipolar protrusive activity or monopolar activity combined with protrusive forces having twice the magnitude. In addition, at the point of optimal fitness, simulated microscopic behaviours appeared to include lateral intercalation as well as radial upward and downward displacements, something which was also observed experimentally^46^. This result suggests that directional cues (for example, radial chemotactic gradient from the EVL) can lead to undirected local migration, introducing an important conceptual difference between cell local displacement as a consequence of biomechanical interactions, and cell polarization axis as a consequence of an externally oriented gradient.

In conclusion, this study highlights MecaGen's ability to integrate collective cell behaviour at the scale of the whole embryo, including custom tissues such as the zebrafish yolk cell and EVL.

## Discussion

The integrative MecaGen platform offers a practical computational framework to test the validity of a wide range of hypotheses about multicellular development and morphogenetic processes both at the genetic/molecular level and at the cellular level of organization. As the above examples show, models and simulations implemented in MecaGen are able to reproduce the collective activity and motion of thousands of cells coordinated by gene regulation and molecular signalling. A central feature is the definition of a simple yet realistic CBO based on three generic cell archetypes of early embryogenesis: mesenchymal, epithelial, idle, and their associated biomechanical behaviours. The core model rules were chosen to represent the most fundamental laws of molecular and cellular dynamics, in order to potentially generate a great diversity of phenomena while keeping the programming effort, computing time and number of parameters reasonable (Supplementary Notes 1 and 2 and Supplementary Tables 2–4). Their combination and distribution over a large assembly of self-organizing elements open up an infinite space of possible emergent phenotypes.

The MecaGen framework was designed to be scalable to integrate new features, but also compatible with massive model exploration and parametric search software (Supplementary Note 3). In particular, combined with evolutionary methods such as genetic algorithms, the developmental machinery of MecaGen contributes to the foundations of a new kind of ‘digital evo-devo' science. Beyond the few case studies presented here, exploitation of a generic model like MecaGen can be applied to a range of morphogenetic events in the embryonic development of different model organisms such as *Xenopus*, *Echinoida*, *Drosophila*, or other types of multicellular processes such as tumour formation, growth and spreading in 3D space and time. We provide the configuration files, tutorials and instructions required to perform the simulations shown here (Supplementary Note 6). Note that the current version does not contain a graphical user interface tool to extend the platform, add new cell types or program a GRN: for now, all customization work must be done by editing directly the source code and configuration files. There is, however, a player utility to visualize simulations in real time. Modellers are invited to reuse, copy or modify the source code distributed under the GNU General Public License v3.0 and freely available at http://www.mecagen.org.

## Methods

### Mathematical model

A comprehensive formal account of the MecaGen model is presented in the Supplementary Information. All equations mentioned in the text can be found there. Supplementary Note 1 defines the cell state variables, which comprise the three cell archetypes (mesenchymal, epithelial, idle) with their associated behaviours, the spatial variables and the genetic variables (equations (1)–(2)). Supplementary Note 2 details the biomechanical component of the model, including the overdamped equation of motion regulating cell displacements, the cell neighbourhoods, the passive and active forces, and the cell cycle rules (equations (3)–(29)). Supplementary Note 3 reviews the principles underlying the cells' chemical activity resting on three rule sets: intracellular gene and protein reactions, ligand secretion and messenger transduction, and extracellular ligand transport and diffusion (equations (30)–(47)). Supplementary Note 4 specifies how both sides are coupled, that is, how the mechanical parameters and archetypes are controlled by the genetic and molecular dynamics, and vice-versa, through a CBO (equations (48)–(58)). The parameter values used in the three case studies are reported in Supplementary Tables 2–4.

### Data availability

MecaGen is an open-source framework available to the scientific community via our project's website http://www.mecagen.org. The source code is hosted at the following GitHub project: https://github.com/juliendelile/MECAGEN.

## Additional information

**How to cite this article:** Delile, J. *et al*. A cell-based computational model of early embryogenesis coupling mechanical behaviour and gene regulation. *Nat. Commun.* **8,** 13929 doi: 10.1038/ncomms13929 (2017).

**Publisher's note**: Springer Nature remains neutral with regard to jurisdictional claims in published maps and institutional affiliations.

## Supplementary Material

Supplementary Information Supplementary Figures, Supplementary Tables, Supplementary Notes and Supplementary References

Supplementary Movie 1 Spatiotemporal dynamics of the patterning study. See Fig. 3 for explanation (same color code, except the intracellular protein Wnt in blue to distinguish it from the extracellular Wnt ligand in red).

Supplementary Movie 2 Spatiotemporal dynamics of the compartment formation study. See Fig. 4 for explanation (same color code). Left: External view of the cellular volume, in which the two epithelial populations are labelled as soon as they appear and reshape themselves. Right: Intratissular slices showing the evolution of each product concentration and the cell movements at the boundary.

Supplementary Movie 3 Spatiotemporal dynamics of the epiboly study. See Fig. 5,6 for explanation (same color code). The movie shows a sagittal section of the complete simulated embryo, from 1548 to 3095 cells. The yolk membrane and particles are displayed in yellow, and the enveloping layer (EVL) particles in purple. The cellular membrane colors represent the concentration gradient of ligand secreted by the EVL.
