# Supplementary material for: A cell-based computational model of early embryogenesis coupling mechanical behaviour and gene regulation
Source: Nat Commun. 2017 Jan 23;8:13929. doi: 10.1038/ncomms13929 (PMC5264012; doi:10.1038/ncomms13929)
Supplement: Supplementary Information — Supplementary Figures, Supplementary Tables, Supplementary Notes and Supplementary References [file ncomms13929-s1.pdf]

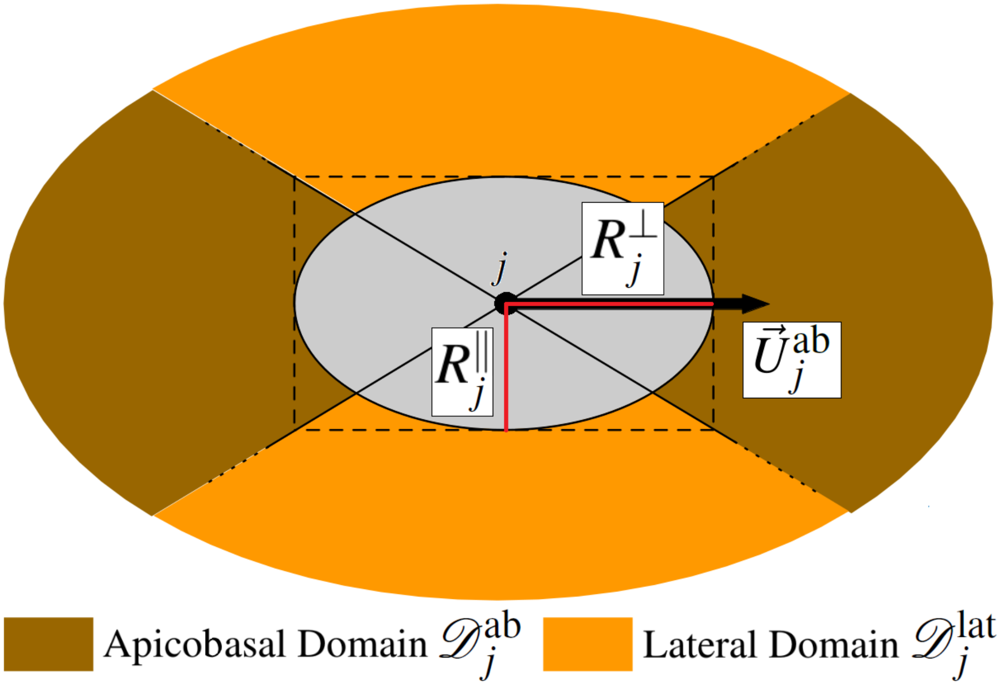

Supplementary Figure 1 | Apicobasal and lateral domains of a nonspherical cell. The AB and lateral domains of a cell j in 3D are separated by a conical surface of axis $\vec{U}_j^{\rm ab}$. The cone's aperture is the angle of one of the sections of $\mathcal{D}_j^{\rm ab}$ in 2D (brown slices). The cosine of half the aperture, denoted by β_j in the text, is determined by the shape of the cell, i.e. its AB radius $R_j^{\rm ab} = R_j^{\perp}$ and lateral radius $R_j^{\rm lat} = R_j^{||}$ (red line segments) according to: $\beta_j = R_j^{\perp}/({R_j^{\perp}}^2 + {R_j^{||}}^2)^{1/2}$. See equation 6.

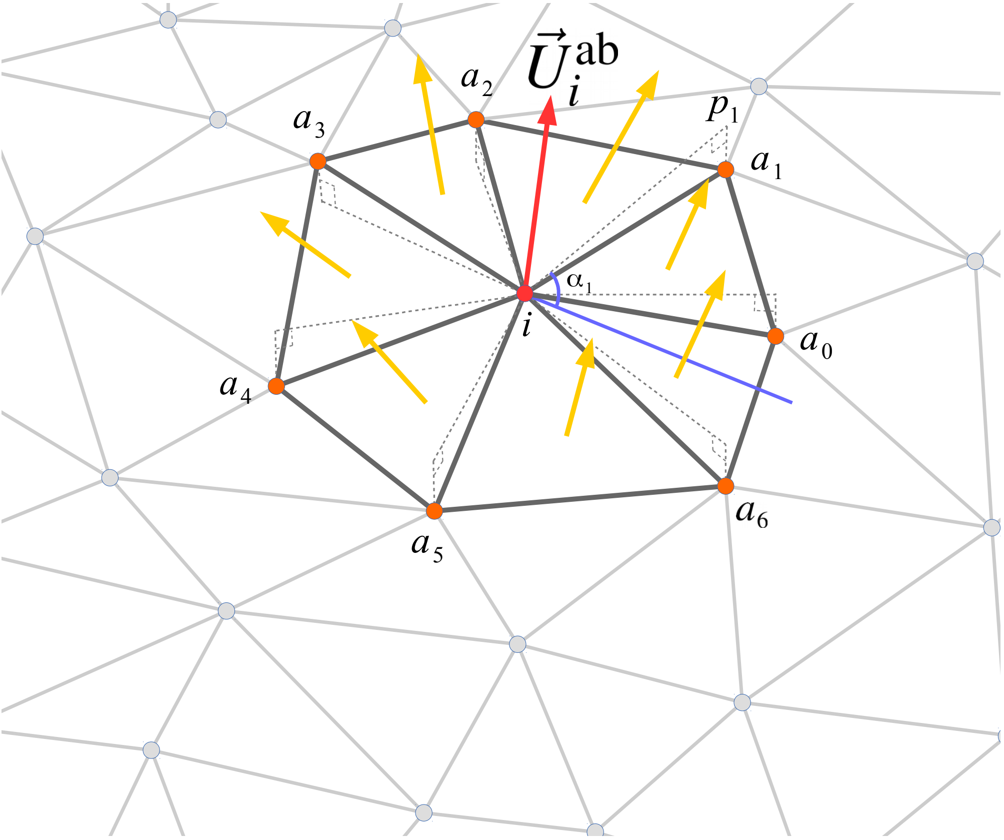

Supplementary Figure 2 | Determination of the apicobasal polarisation axis. The AB axis of cell i, $\vec{U}_i^{\mathrm{ab}}$ (red arrow) is calculated as the average of the outward vectors (yellow arrows), which are orthogonal to the triangular facets made of neighbourhood edges (grey lines). To find the surrounding triangles, neighbour positions are first projected on the plane orthogonal to the current axis $\vec{U}_i^{\mathrm{ab}}$ (e.g. a_1 becomes p_1 here), then these positions are sorted according to the angles α_j formed by the vectors (i, p_j) with an arbitrary line in the orthogonal plane (blue line). See equation 9.

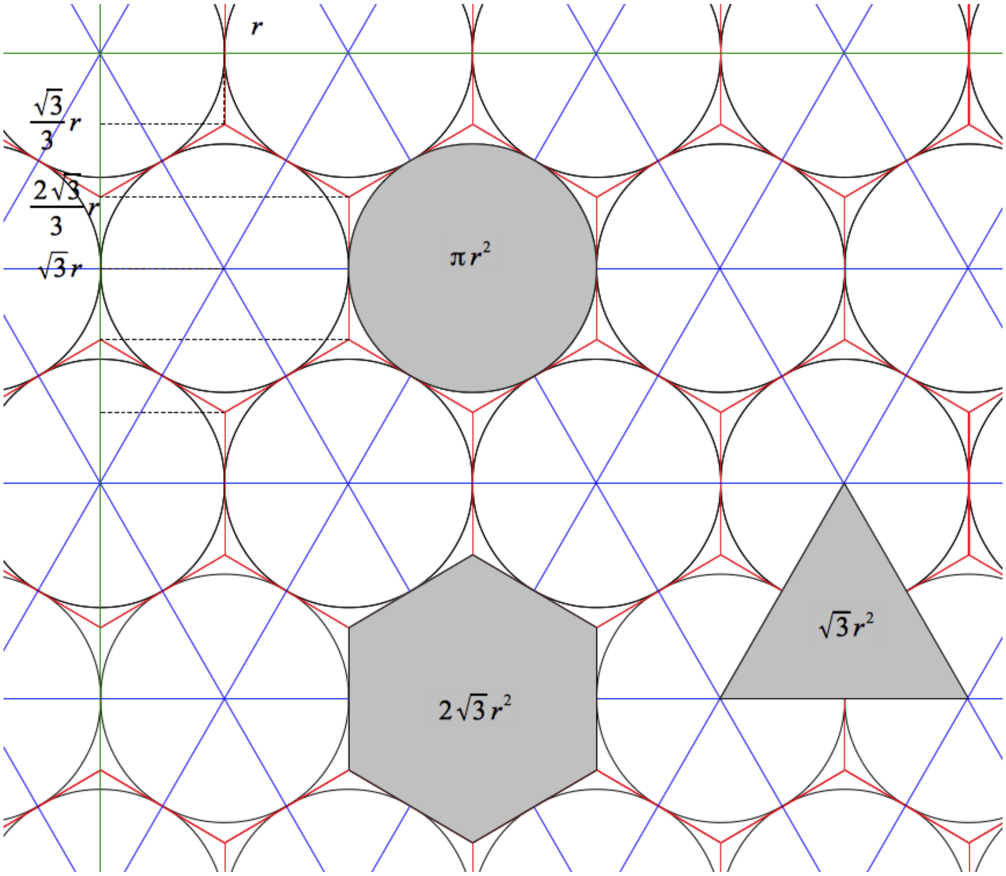

Supplementary Figure 3 | Geometrical properties of the hexagonal lattice. In the 2D plane, the hexagonal lattice realises the densest packing of equal-radius disks. See equation 11.

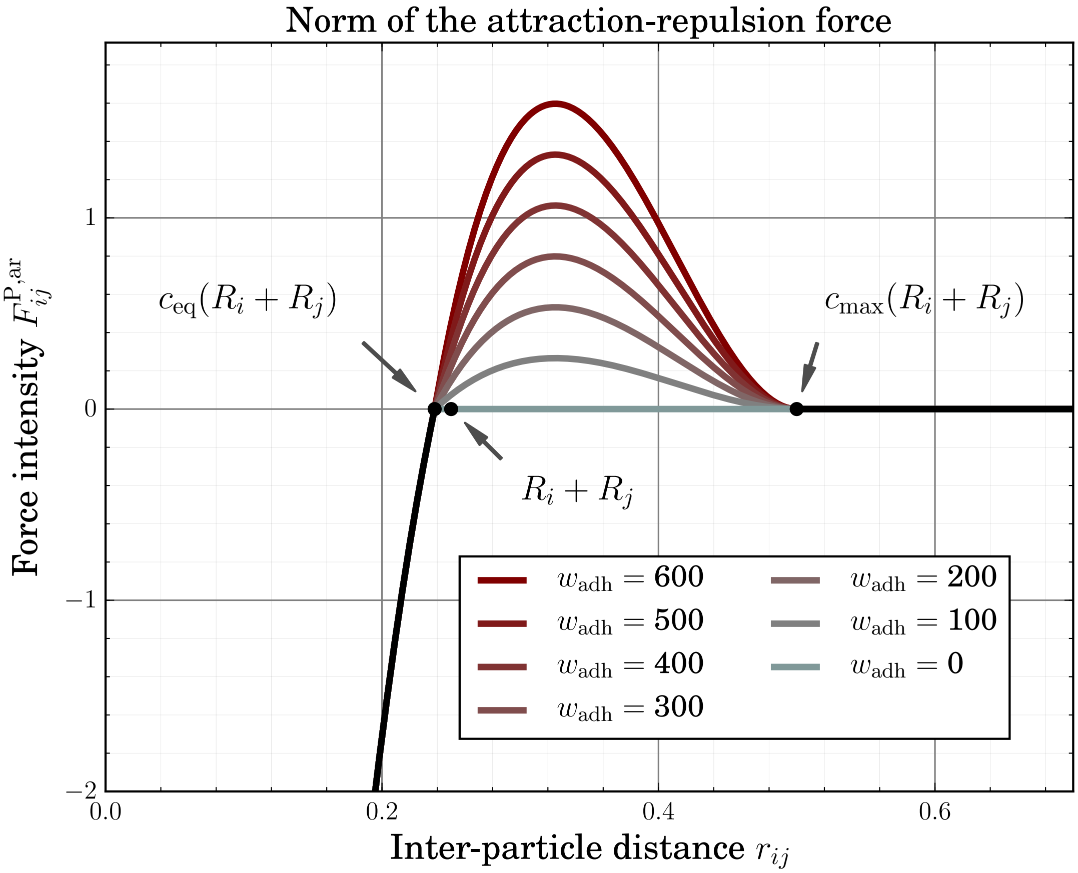

Supplementary Figure 4 | Profile of the attraction-repulsion force. The plot of $\vec{F}_{ij}^{\mathrm{P,ar}}$ is composed of three distance intervals: repulsion (negative force) at short range until $r_{ij}^{\mathrm{eq}} = c_{\mathrm{eq}}(R_i + R_j)$; attraction (positive force) at mid-range between r_{ij}^{eq} and $r_{ij}^{\mathrm{max}} = c_{\mathrm{max}}(R_i + R_j)$; shown here under different values of the adhesion coefficient w_{adh}; and neutrality (zero force) at long range beyond r_{ij}^{max}. See equation 14.

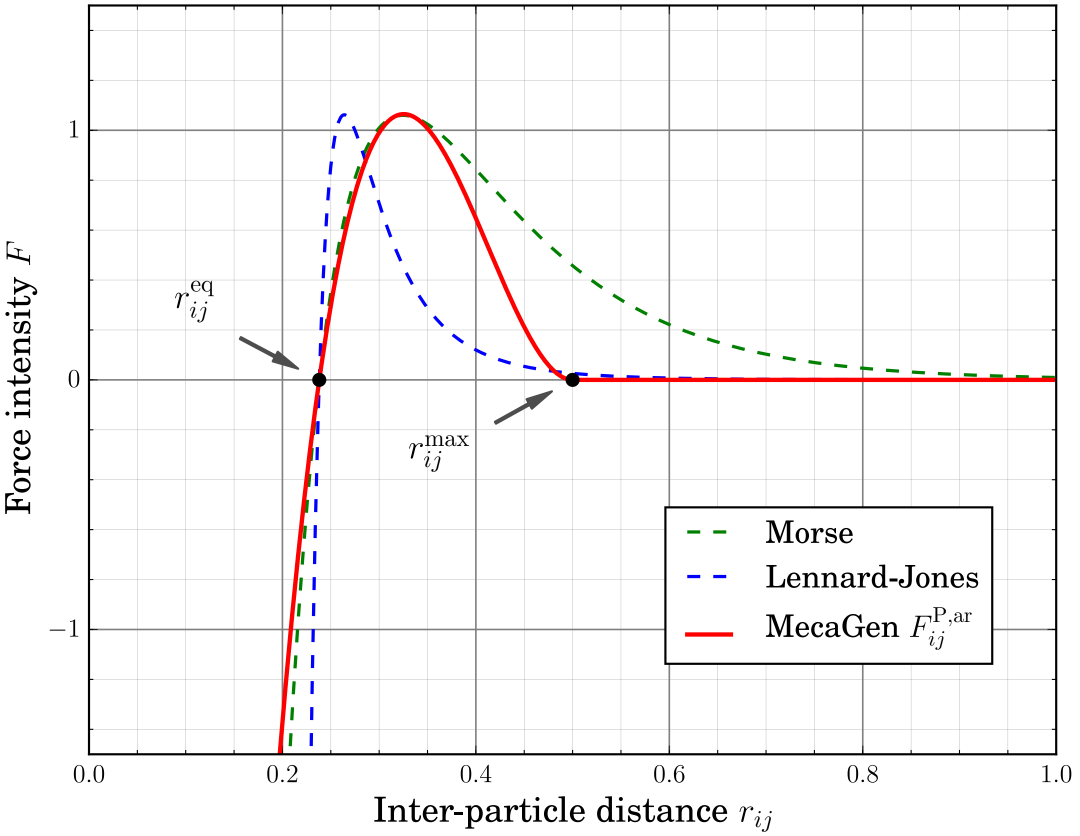

Supplementary Figure 5 | Comparison of the attraction-repulsion force with classical laws. With $R_i = 0.1$, $R_j = 0.15$ and $c_{\mathrm{eq}} = 0.9523$, the equilibrium distance is set to $r_{ij}^{\mathrm{eq}} = c_{\mathrm{eq}}(R_i + R_j) = 0.238$. Solid red line: AR potential $\vec{F}_{ij}^{\mathrm{P,ar}}$ with $w_{\mathrm{adh}} = w_{\mathrm{rep}} = 400$ (equation 14). Dashed green line: Force derived from the Morse potential of equation $2\delta K(e^{-K(r-r_{ij}^{\mathrm{eq}})} - e^{-2K(r-r_{ij}^{\mathrm{eq}})})$, with $\delta = 0.265$ and $K = 8$. Dashed blue line: Force derived from the Lennard-Jones potential of equation: $-24(\varepsilon/\sigma)(2(r/\sigma)^{-13} - (r/\sigma)^{-7})$, with $\varepsilon = 0.094$ and $\sigma = 2^{-1/6} r_{ij}^{\mathrm{eq}}$. Parameters were tuned to make the three equilibrium distances coincide in r_{ij}^{eq} and the three maximum values match approximately.

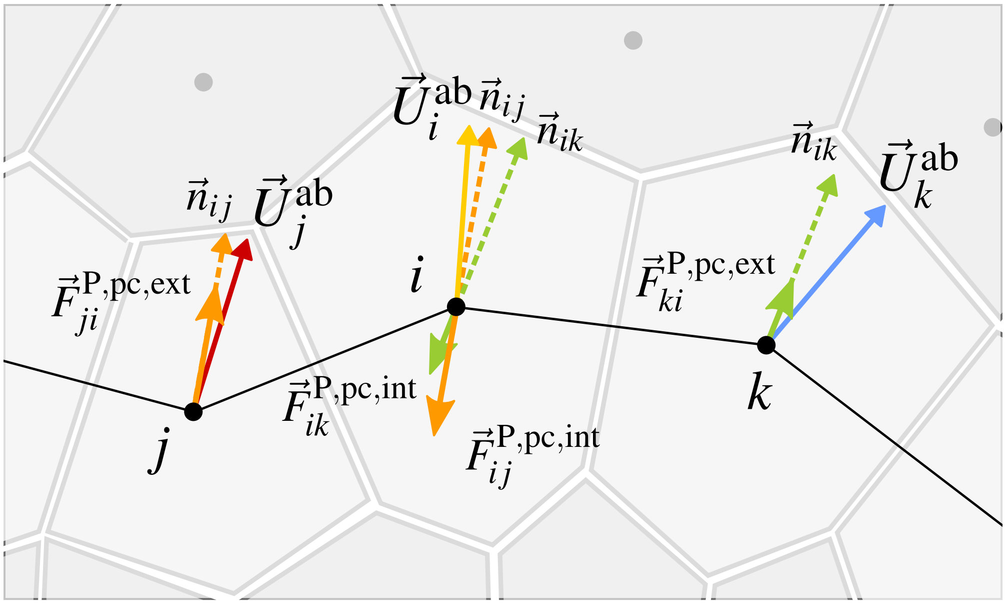

Supplementary Figure 6 | Planarity conservation forces between neighbouring epithelial cells. For the sake of the illustration, cells i, j and k are positioned in the same vertical section. Their polarisation axes are $\vec{U}_i^{\mathrm{ab}}$ (yellow arrow), $\vec{U}_j^{\mathrm{ab}}$ (red arrow) and $\vec{U}_k^{\mathrm{ab}}$ (blue arrow), respectively. The PC forces exerted by neighbouring cells i and j on each other (thick orange arrows), and by i and k on each other (short thick green arrows), are parallel to the shared edge-AB vectors $\vec{n}_{ij}$ (dashed orange arrows) and $\vec{n}_{ik}$ (dashed green arrow), respectively. Force intensity is less between i and k because $\vec{n}_{ik}$ and the edge vector $\vec{u}_{ik}$ are nearly orthogonal. As a result of these forces, neighbouring cells are always attracted back to the lateral domain of each cell and thus tend to maintain the planarity of the tissue. See equations 15-17.

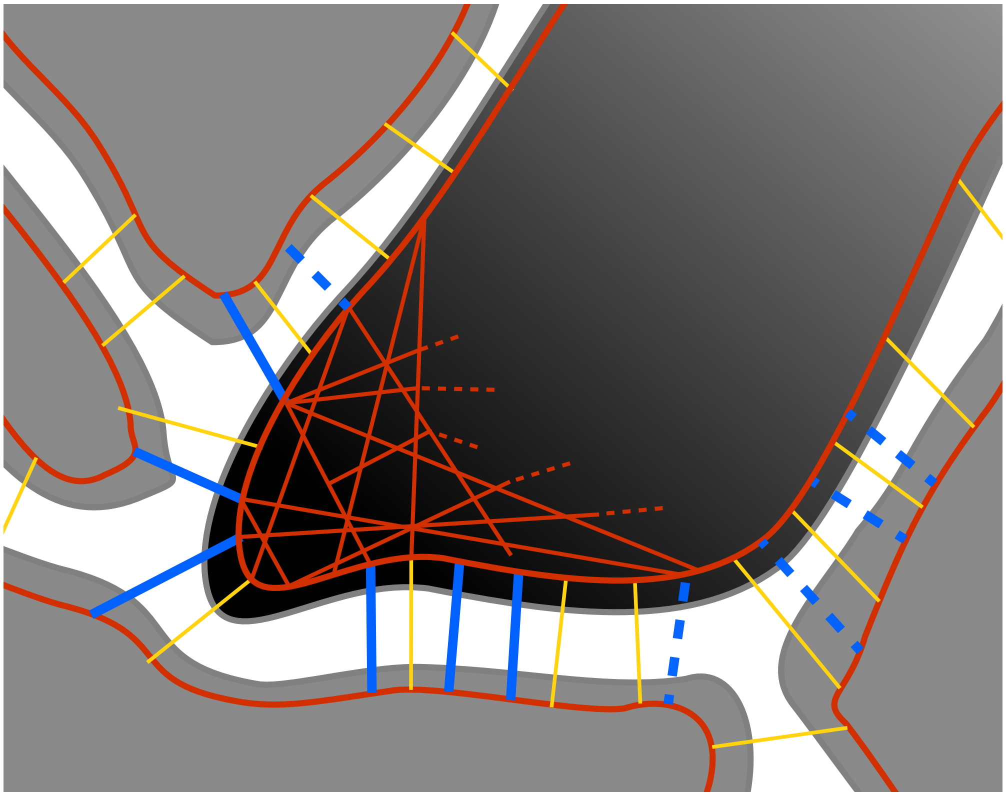

Supplementary Figure 7 | Diagram of cell protrusion with subcellular elements. A cell is attached to its neighbours by molecular bridges (here symbolised by thin yellow edges), which actively deform their internal actomyosin cortical network (red lines and curves) in the direction of the polarisation axis indicated by the internal chemical gradient (dark gray shades). A bulge eventually appears at the active pole, pushing away neighbouring cells. No intercalation process would be observed, however, without a precise regulation of the adhesion contacts between the cells. Thus, in addition to the regular adhesion bonds (yellow), special *focal adhesion points* (thick blue edges) also appear at the surface of the protruding region of the cell. These bonds bear the extra load generated by the protrusive activity of the actomyosin cortex. They become visible around the tip of the bulge and, as the cell is advancing, maintain spatial cohesion between neighbouring cytoskeletons. Without them, the cell would slip on the surface and the efficiency of the protrusion would be greatly reduced. Focal adhesion points gradually disappear from the cell membrane (dashed blue) as the cell advances relatively to the bonded neighbours.

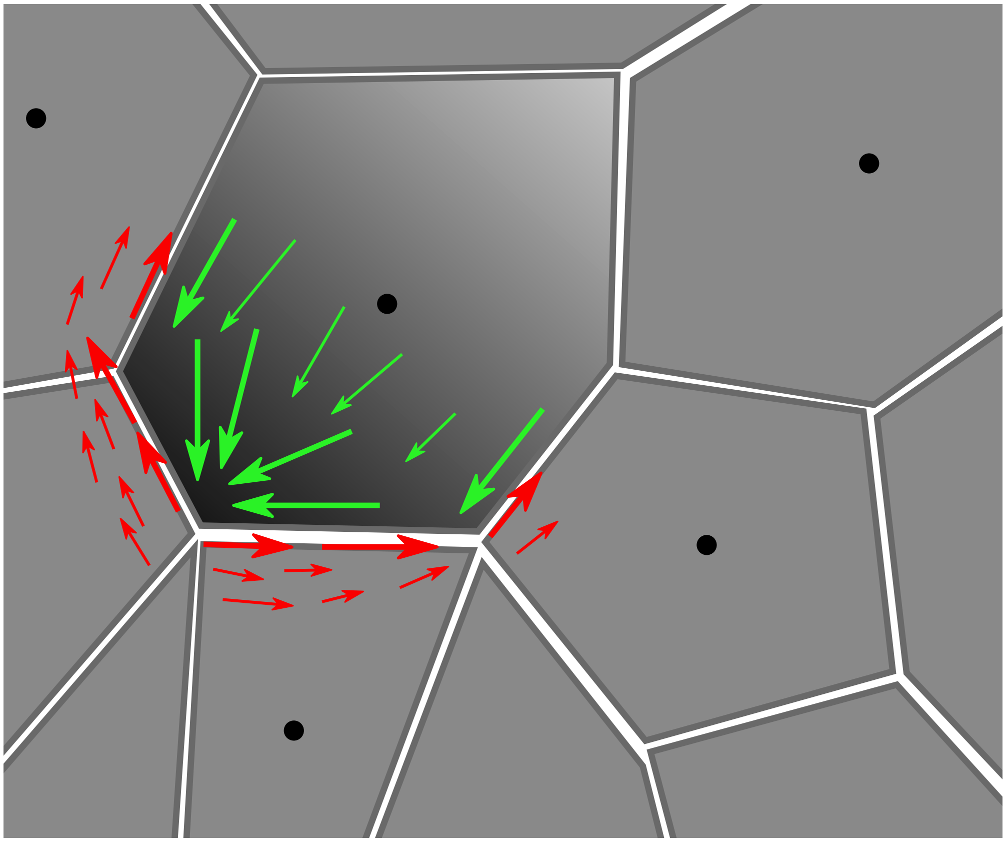

Supplementary Figure 8 | Idealised diagram of cell protrusion. Green arrows represent the cell interior flow and red arrows the cell neighbourhood flow, as the central cell exerts a protrusion over its surroundings. The schematised focal adhesion points of Supplementary Fig. 7, which appear at the tip of the cell (but not shown here), move back then disappear as the cell moves forward.

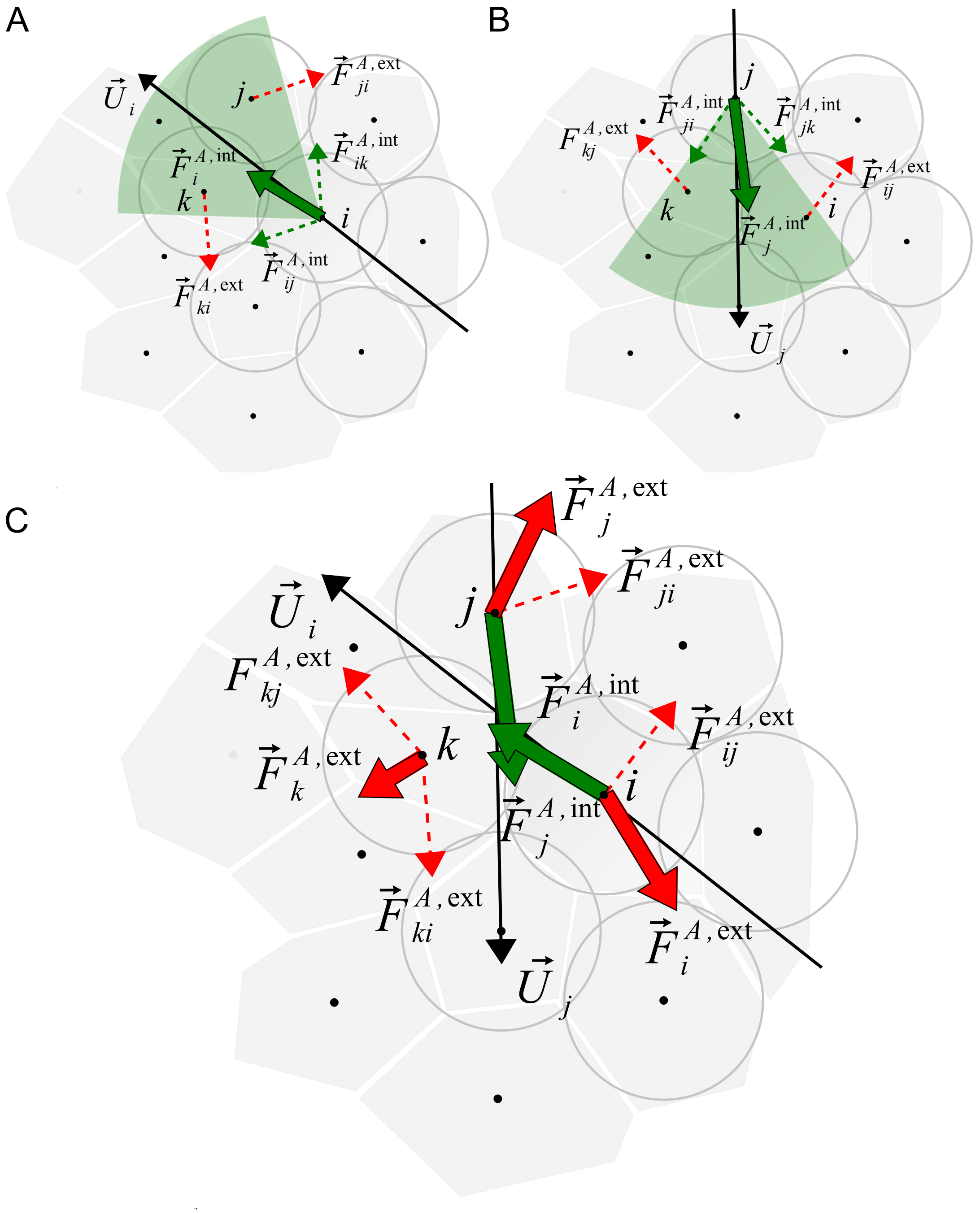

Supplementary Figure 9 | Schematic force model of cell protrusion. Formalisation of the idealised mechanism of Supplementary Fig. 8 in the particle-based framework. A: Forces produced by i's activity. The polar domain (green slice) of cell i, denoted by $\mathcal{N}_i^+$ in the text, contains two neighbour cells j and k, over which i exerts a protrusive force. "Intrinsic" forces are shown in green and extrinsic forces in red. B: Forces produced by j's activity if j, too, happens to be protruding. C: The net resulting "active" forces (not shown) are obtained by adding the net resulting 'int' (thick green) and 'ext' (thick red) arrows on each cell. See equations 19-21.

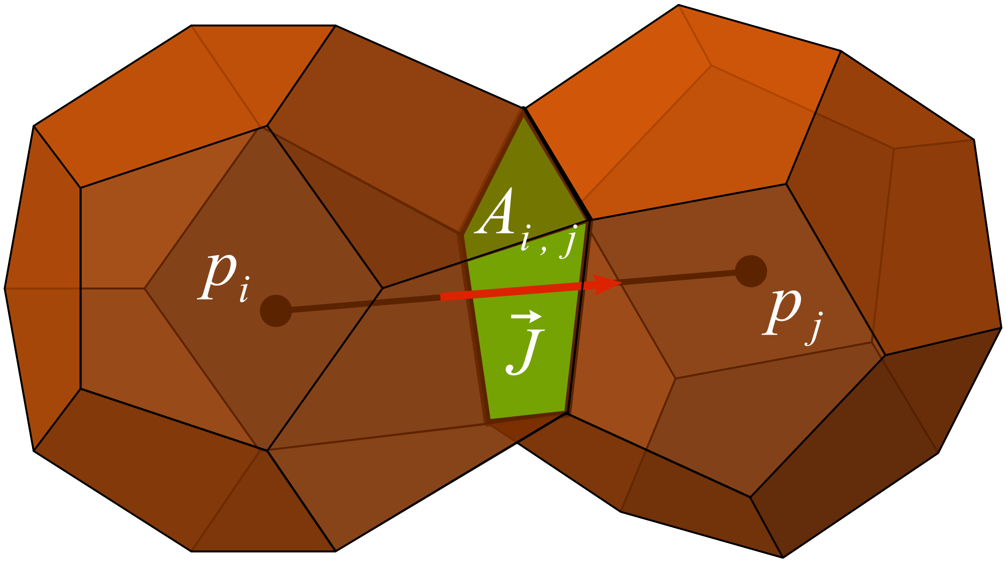

Supplementary Figure 10 | Schema of extracellular ligand diffusion. Here, $\vec{J}$ (red arrow) represents the unidirectional flux of ligand Q_c between the cellular volumes occupied by neighbouring cells i and j (brown polyhedra), and A_{ij} is the contact area (green polygon): $\vec{J}_{c,ij} = \vec{J}_{c,ji} = -D_c(q_{c,j} - q_{c,i})\vec{u}_{ij}/r_{ij}$. See equation 44.

Supplementary Table 1a | Historical and comparative review of models of biological development.
Features (a-d): type of model, mechanical rules, chemical rules, coupling rules (see Supplementary References).

Year	Authors	Title	(a) Multi-agent / Macroscopic	(b) Mechanics	(c) Molecular Signaling and/or Genetic Regulation	(d) Coupling
1917 (1942)	Thompson, D.W. [1]	*On growth and form*	macroscopic: describes geometrical transformations to relate animal forms	no	no	--
1952	Turing, A.M. [2]	The chemical basis of morphogenesis	macroscopic: reaction-diffusion of morphogens in continuous space	no	yes	--
1969	Wolpert, L. [3]	Positional information and the spatial pattern of cellular differentiation	macroscopic: gradient-induced fields and polarity	no	yes	--
1972	Gierer, A. & Meinhardt, H. [4]	A theory of biological formation	macroscopic: pattern formation, continuous space, reactions combining self-activation and long-range inhibition	no	yes	--
1990	Weliky, M. & Oster, G. [5]	The mechanical basis of cell rearrangement. I. Epithelial morphogenesis during Fundulus epiboly	vertex-based	yes: 2D polygons, internal pressure, damped equation of motion	no	--
1991	Weliky, M., Minsuk, S., Keller, R. & Oster, G. [6]	Notochord morphogenesis in Xenopus laevis: simulation of cell behavior underlying tissue convergence and extension	vertex-based	yes: 2D polygons, internal pressure, damped equation of motion	no	--
1991	Mjolsness, E., Sharp, D.H. & Reinitz, J. [7]	A connectionist model of development (Cellerator)	multi-agent	yes: simple, user-defined external forces	yes: reaction + diffusion toward neighbors	--
1993	Glazier, J. & Graner, F. [8]	Simulation of the differential adhesion driven rearrangement of biological cells.	first cellular Potts model (CPM)	yes	no	--
2001	Shapiro, B.E. & Mjolsness, E.D. [9]	Developmental simulations with Cellerator	multi-agent	particle-based elastic forces	yes: chemical reactions (ODEs)	yes
2001	Marée, A.F.M. & Hogeweg, P. [10]	How amoeboids self-organize into a fruiting body: multicellular coordination in Dictyostelium discoideum	CPM	yes	yes: two-variable FitzHugh-Nagumo eqs, piecewise linear "Pushchino kinetics" for oscillating cAMP waves	yes: adds chemotactic potential from local cAMP gradient to Hamiltonian
2002	Meir, E.L.I., Munro, E.M., Odell, G.M. & Von Dassow, G. [11]	Ingeneue: A versatile tool for reconstituting genetic networks, with examples from the segment polarity network	multi-agent: GRN is instantiated in each cell	static cells	intracellular ODEs + multicompartment (intra and surface proteins) + cell-cell communication + diffusion	--
2004	Dallon, J.C. & Othmer, H.G. [12]	How cellular movement determines the collective force generated by the Dictyostelium discoideum slug	center-based, ellipsoids	yes	no	--
2005	Schaller, G. & Meyer-Hermann, M. [13]	Multicellular tumor spheroid in an off-lattice Voronoi-Delaunay cell model	multi-agent, center-based, topological neighborhood	yes	yes: metabolic environment of the tumor	yes: defines cell state
2007	Robertson, S.H. *et al.* [14]	Multiscale computational analysis of Xenopus laevis morphogenesis reveals key insights of systems-level behavior	agent-based modeling (ABM), NetLogo	on-lattice but not CPM: agents can overlap on 2D pixels to emulate 3D	yes: intracellular reactions	--
2007	Käfer, J., Hayashi, T., Marée, A.F.M., Carthew, R.W. & Graner, F. [15]	Cell adhesion and cortex contractility determine cell patterning in the Drosophila retina	CPM: adding cell cortical contraction to adhesion-driven surface increase	yes	no	--
2008	Krieg, M. *et al.* [16]	Tensile forces govern germ-layer organization in zebrafish	CPM	yes	no	--
2008	Honda, H., Motosugi, N., Nagai, T., Tanemura, M. & Hiiragi, T. [17]	Computer simulation of emerging asymmetry in the mouse blastocyst	vertex-based: damped equation of motion, surface energy, volume conservation	yes	no	--
2008	Rejniak, K.A. & Anderson, A.R.A. [18]	A computational study of the development of epithelial acini: I. Sufficient conditions for the formation of a hollow structure	both vertex-based and continuous for the fluid (Navier-Stokes)	yes: elastic particles for cell membrane + viscous fluid inside and outside the cell	no: cell type is specified by ad hoc rule	--
2010	Hoehme, S. & Drasdo, D. [19]	(CellSys) A cell-based simulation software for multi-cellular systems	center-based (spheroid)	JKR force model	diffusion and nutrient consumption on a lattice	--
2011	Sandersius, S.A., Chuai, M., Weijer, C.J. & Newman, T.J. [20]	Correlating cell behavior with tissue topology in embryonic epithelia	Subcellular Element Model (ScEM): multi-particle cells, no difference between membrane and inner cell particles	yes: Morse potential	yes	substance concentration levels induce cell growth, and mitosis if threshold is reached (size threshold)
2011	Hester, S. D. *et al.* [21]	A multi-cell, multi-scale model of vertebrate segmentation and somite formation	CPM + genetic regulation (using CompCell3D)	yes	yes: intracellular + communication + diffusion	Boolean cell type determination network
2011	Sandersius, S.A., Chuai, M., Weijer, C.J. & Newman, T.J. [22]	A "chemotactic dipole" mechanism for large-scale vortex motion during primitive streak formation in the chick embryo	Subcellular Element Model (ScEM): multi-particle cells, no difference between membrane and inner cell particles	yes: includes migration axes induced by mechanotaxis	yes	linear diffusion with Poisson-noise degradation field inducing polarization axis for chemotactic behavior
2011	Merks, R.M.H., Guravage, M., Inzé, D. & Beemster, G.T.S. [23]	VirtualLeaf: an open-source framework for cell-based modeling of plant tissue growth and development	vertex-based	Hamiltonian energy, MonteCarlo simulation	transport of chemical species	yes
2013	Shapiro, B.E., Meyerowitz, E.M. & Mjolsness, E. [24]	Using Cellzilla for plant growth simulations at the cellular level	vertex-based	Hookean springs + cell internal pressure	intracellular ODEs (wide range of interaction models) + diffusion	--
2015	Tanaka, S., Sichau, D. & Iber, D. [25]	LBIBCell: a cell-based simulation environment for morphogenetic problems	vertex-based	immersed boundary method	reaction-advection-diffusion (PDEs, Lattice Boltzmann method)	yes: cell type determined by signaling factor concentration

Supplementary Table 1b | Historical and comparative review of models of biological development. Features (e-i): cell behaviours, cell division, species and structures, data, 3D (see Supplementary References).

Year	Authors	Title	(e) Cell Behaviors	(f) Cell Division	(g) Species and Structures	(h) Compared with Data	(i) 3D
1917 (1942)	Thompson, D.W. [1]	*On growth and form*	--	no	various	no	no
1952	Turing, A.M. [2]	The chemical basis of morphogenesis	--	no	tentacles in *Hydra*	no	no
1969	Wolpert, L. [3]	Positional information and the spatial pattern of cellular differentiation	tissue growth	yes (macroscopic continuum)	early devel. of sea urchin, regeneration of hydroids,	no	no
1972	Gierer, A. & Meinhardt, H. [4]	A theory of biological formation	polarization in a gradient field	no	*Hydra*	no	no
1990	Weliky, M. & Oster, G. [5]	The mechanical basis of cell rearrangement. I. Epithelial morphogenesis during Fundulus epiboly	migration towards vegetal pole (epiboly)	no	*Fundulus*	no	no: 2D on a sphere
1991	Weliky, M., Minsuk, S., Keller, R. & Oster, G. [6]	Notochord morphogenesis in Xenopus laevis: simulation of cell behavior underlying tissue convergence and extension	polarization, intercalation	no	*Xenopus laevis*	no	no
1991	Mjolsness, E., Sharp, D.H. & Reinitz, J. [7]	A connectionist model of development (Cellerator)	differentiation	yes: grammar rules	blastoderm of *Drosophila*	no	no
1993	Glazier, J. & Graner, F. [8]	Simulation of the differential adhesion driven rearrangement of biological cells.	cell sorting, cell dispersal	no	no	no	no
2001	Shapiro, B.E. & Mjolsness, E.D. [9]	Developmental simulations with Cellerator	cell growth, cell death	yes	no	no	yes
2001	Marée, A.F.M. & Hogeweg, P. [10]	How amoeboids self-organize into a fruiting body: multicellular coordination in Dictyostelium discoideum	chemotaxis	no	*Dictyostelium discoideum*	no	no
2002	Meir, E.L.I., Munro, E.M., Odell, G.M. & Von Dassow, G. [11]	Ingeneue: A versatile tool for reconstituting genetic networks, with examples from the segment polarity network	no	no	segment polarity in *Drosophila* (associated publication)	no	no
2004	Dallon, J.C. & Othmer, H.G. [12]	How cellular movement determines the collective force generated by the Dictyostelium discoideum slug	collective migration	no	*Dictyostelium discoideum*	no	yes
2005	Schaller, G. & Meyer-Hermann, M. [13]	Multicellular tumor spheroid in an off-lattice Voronoi-Delaunay cell model	mitosis, apoptosis (and tissue growth)	yes: grammar rules	tumors	yes	no
2007	Robertson, S.H. *et al.* [14]	Multiscale computational analysis of Xenopus laevis morphogenesis reveals key insights of systems-level behavior	migration on fibronectin matrix	no: 53 cells	mesendoderm migration in *Xenopus laevis*	no	no
2007	Käfer, J., Hayashi, T., Marée, A.F.M., Carthew, R.W. & Graner, F. [15]	Cell adhesion and cortex contractility determine cell patterning in the Drosophila retina	cell reshaping	no	retina of *Drosophila*	not automated: visual comparison	no
2008	Krieg, M. *et al.* [16]	Tensile forces govern germ-layer organization in zebrafish	cell sorting	no	zebrafish	no	no
2008	Honda, H., Motosugi, N., Nagai, T., Tanemura, M. & Hiiragi, T. [17]	Computer simulation of emerging asymmetry in the mouse blastocyst	mechanical evolution of cell shapes	no: 40 cells	mouse blastocyst	no	yes
2008	Rejniak, K.A. & Anderson, A.R.A. [18]	A computational study of the development of epithelial acini: I. Sufficient conditions for the formation of a hollow structure	epithelial polarization, apoptosis, growth	yes	epithelial acini	no	no
2010	Hoehme, S. & Drasdo, D. [19]	(CellSys) A cell-based simulation software for multi-cellular systems	cell growth, migration	yes	liver regeneration (other publication)	no	yes
2011	Sandersius, S.A., Chuai, M., Weijer, C.J. & Newman, T.J. [20]	Correlating cell behavior with tissue topology in embryonic epithelia	cell growth	yes	hydra, *Drosophila*, *Xenopus*, chick	yes: no fitness	no
2011	Hester, S. D. *et al.* [21]	A multi-cell, multi-scale model of vertebrate segmentation and somite formation	cell motility controlled by a parameter	no: adds cells at the posterior end of the presomitic mesoderm	vertebrate somitogenesis	no	no
2011	Sandersius, S.A., Chuai, M., Weijer, C.J. & Newman, T.J. [22]	A "chemotactic dipole" mechanism for large-scale vortex motion during primitive streak formation in the chick embryo	chemotaxis, mechanotaxis	no: 1200 cells	primitive streak formation in the chick embryo	qualitative	no
2011	Merks, R.M.H., Guravage, M., Inzé, D. & Beemster, G.T.S. [23]	VirtualLeaf: an open-source framework for cell-based modeling of plant tissue growth and development	cell growth	yes	*Arabidopsis* (plant) in other publications	no	no
2013	Shapiro, B.E., Meyerowitz, E.M. & Mjolsness, E. [24]	Using Cellzilla for plant growth simulations at the cellular level	cell growth	yes	plants in other publications	no	no
2015	Tanaka, S., Sichau, D. & Iber, D. [25]	LBIBCell: a cell-based simulation environment for morphogenetic problems	cell growth	yes	no	no	no

Supplementary Table 2 | Parameter values of the patterning study. See Fig. 3. Row titles and equation numbers refer to the corresponding sections and equations. Time step: $\Delta t = 6$ s.

**Mechanical Rules**	$R = 10$ (eq. 1,2), $\lambda = 3000$ (eq. 3), $a = 1.3697$ (eq. 4)<br>$c_{\max} = 1.6$ (eq. 5), $c_{\text{eq}} = 0.9523$ (eq. 12), $w_{\text{rep}} = w_{\text{adh}} = 100$ (eq. 14)<br>$T_0 = 10\Delta t$, $r_0 = 1.5 \pm 0.5$ (eq. 24), $\mu = 0.5$ (eq. 28), $\eta = 0.15$ (eq. 29),
**Gene Expression** (eq. 30,31)	*Target* $\begin{cases} \theta_{\text{Tcf}+} = \theta_{\text{Tcf}-} = 30 \\ f_{Tar} = \Gamma_{\text{Tcf}+} \text{ AND NOT } \Gamma_{\text{Tcf}-} \end{cases}$
**Protein Synthesis** (eq. 32)	Target $\{\gamma = 0.1$
**Protein Reactions** (eq. 34,35)	β-cat + Tcf $\to$ Tcf+ $\begin{cases} \alpha_{\beta\text{-cat}} = 0,\ x_{\beta\text{-cat}} = 1 \\ \alpha_{\text{Tcf}} = 0,\ x_{\text{Tcf}} = 1 \\ \alpha_{\text{Tcf}+} = 1,\ k = 2\text{E-5} \end{cases}$<br>Gro + Tcf $\to$ Tcf− $\begin{cases} \alpha_{\text{Gro}} = 0,\ x_{\text{Gro}} = 1 \\ \alpha_{\text{Tcf}} = 0,\ x_{\text{Tcf}} = 1 \\ \alpha_{\text{Tcf}-} = 1,\ k = 2\text{E-5} \end{cases}$<br>XIAP + Gro $\to$ Gro^{Ubi} $\begin{cases} \alpha_{\text{XIAP}} = 1,\ x_{\text{XIAP}} = 1 \\ \alpha_{\text{Gro}} = 1,\ x_{\text{Gro}} = 1 \\ \alpha_{\text{Gro}^{\text{Ubi}}} = 1,\ k = 4\text{E-5} \end{cases}$
**Protein Degradation** (eq. 36)	Wnt $\{\kappa = 0.005,$ β-cat $\{\kappa = 0.0067$<br>XIAP, Tcf, Gro, Tcf+, Tcf−<br>Target, Gro^{Ubi}, Fzd, XIAP-ind $\{\kappa = 0.0017$
**Protein Programming** (eq. 38)	Wnt $\begin{cases} \gamma = 1.3,\ [t_{\min}, t_{\max}] = [20, +\infty) \\ [x_{\min}, x_{\max}] = [y_{\min}, y_{\max}] = (-\infty, 0] \\ [z_{\min}, z_{\max}] = (-\infty, +\infty) \end{cases}$<br>Tcf, Gro<br>Fzd, XIAP-ind $\begin{cases} \gamma = 0.1,\ [t_{\min}, t_{\max}] = [0, +\infty) \\ [x_{\min}, x_{\max}] = [y_{\min}, y_{\max}] = [z_{\min}, z_{\max}] = (-\infty, +\infty) \end{cases}$
**Ligand Secretion** (eq. 39)<br>**Ligand Degradation** (eq. 42)<br>**Ligand Diffusion** (eq. 44)	Wnt-lig $\begin{cases} \sigma = 0.0167 \\ \chi = 0.02 \\ D = 0.005 \end{cases}$
**Signal Transduction**<br>Diffusive ligand (eq. 40)	Wnt-lig + Fzd $\to$ β-cat $\begin{cases} \alpha_{\text{Wnt-lig}} = 0,\ x_{\text{Wnt-lig}} = 1 \\ \alpha_{\text{Fzd}} = 0,\ x_{\text{Fzd}} = 1 \\ \alpha_{\beta\text{-cat}} = 1,\ \tau = 1\text{E-4} \end{cases}$<br>Wnt-lig + XIAP-ind $\to$ XIAP $\begin{cases} \alpha_{\text{Wnt-lig}} = 0,\ x_{\text{Wnt-lig}} = 1 \\ \alpha_{\text{XIAP-ind}} = 0,\ x_{\text{XIAP-ind}} = 1 \\ \alpha_{\text{XIAP}} = 1,\ \tau = 3\text{E-5} \end{cases}$

Supplementary Table 3 | Parameter values of the compartment formation study. See Fig. 4. Row titles and equation numbers refer to the corresponding sections and equations. Time step: $\Delta t = 6$ s.

**Mechanical Rules**	$R = R^{\parallel} = 10,\ R^{\perp} = 15$ (eq. 1,2), $\lambda = 3000$ (eq. 3)<br>$a = 1.3697$ (eq. 4), $c_{\text{max}} = 1.6$ (eq. 5), $c_{\text{eq}} = 0.9523$ (eq. 12)<br>$w_{\text{rep}} = w_{\text{adh}} = 100$ (eq. 14), $k_{\text{rig}} = 4000$ (eq. 17)
**Gene Expression** (eq. 30,31)	*Ant* $\begin{cases} \theta_{\text{X}} = 1,\ \theta_{\text{Ant}} = 10 \\ f_{Ant} = \Gamma_{\text{X}}\ \text{OR}\ \Gamma_{\text{Ant}} \end{cases}$<br>*Delta* $\begin{cases} \theta_{\text{Ant}} = 30 \\ f_{Delta} = \Gamma_{\text{Ant}} \end{cases}$<br>*Delto* $\begin{cases} \theta_{\text{Epi}} = 90 \\ f_{Delto} = \Gamma_{\text{Epi}} \end{cases}$<br>*Epi* $\begin{cases} \theta_{\text{Epi-ind}} = \theta_{\text{Ant}} = \theta_{\text{Epi}} = 10 \\ f_{Epi} = (\Gamma_{\text{Epi-ind}}\ \text{AND NOT}\ \Gamma_{\text{Ant}})\ \text{OR}\ \Gamma_{\text{Epi}} \end{cases}$<br>*Epi2* $\begin{cases} \theta_{\text{Epi2-ind}} = \theta_{\text{Ant}} = \theta_{\text{Epi2}} = 10 \\ f_{Epi2} = (\Gamma_{\text{Epi2-ind}}\ \text{AND}\ \Gamma_{\text{Ant}})\ \text{OR}\ \Gamma_{\text{Epi2}} \end{cases}$
**Protein Synthesis** (eq. 32)	Ant, Delta, Delto, Epi, Epi2 $\{\gamma = 1.5$
**Protein Degradation** (eq. 36)	X, Ant, Delta, Delto, Epi, Epi2, Epi-ind, Epi2-ind, Notch $\{\kappa = 0.015$
**Protein Programming** (eq. 38)	X $\begin{cases} \gamma = 1,\ [t_{\text{min}}, t_{\text{max}}] = [20, 21] \\ [x_{\text{min}}, x_{\text{max}}] = (-\infty, 0] \\ [y_{\text{min}}, y_{\text{max}}] = [z_{\text{min}}, z_{\text{max}}] = (-\infty, +\infty) \end{cases}$<br>Notch $\begin{cases} \gamma = 1,\ [t_{\text{min}}, t_{\text{max}}] = [0, +\infty) \\ [x_{\text{min}}, x_{\text{max}}] = [y_{\text{min}}, y_{\text{max}}] = [z_{\text{min}}, z_{\text{max}}] = (-\infty, +\infty) \end{cases}$
**Ligand Secretion** (eq. 39)<br>**Ligand Degradation** (eq. 42)<br>**Ligand Diffusion** (eq. 44)	Delta-lig, Delto-lig $\begin{cases} \sigma = 0.015 \\ \chi = 0.02 \\ D = 0 \end{cases}$
**Signal Transduction**<br>Nondiffusive ligand (eq. 41)	Delta-lig + Notch → Epi-ind $\begin{cases} \alpha_{\text{Delta-lig}} = 0,\ x_{\text{Delta-lig}} = 1 \\ \alpha_{\text{Notch}} = 0,\ x_{\text{Notch}} = 1 \\ \alpha_{\text{Epi-ind}} = 1,\ \tau = 1\text{E-3} \end{cases}$<br>Delto-lig + Notch → Epi2-ind $\begin{cases} \alpha_{\text{Delto-lig}} = 0,\ x_{\text{Delto-lig}} = 1 \\ \alpha_{\text{Notch}} = 0,\ x_{\text{Notch}} = 1 \\ \alpha_{\text{Epi2-ind}} = 1,\ \tau = 1\text{E-3} \end{cases}$
**Control of Archetype** (eq. 30,31)	Epithelial $\begin{cases} \theta_{\text{Epi}} = \theta_{\text{Epi2}} = 70 \\ f_{\mathcal{E}} = \Gamma_{\text{Epi}}\ \text{OR}\ \Gamma_{\text{Epi2}} \end{cases}$
**Control of Polarisation** (eq. 30,31)<br>Chemotactic mode (eq. 52)	$m = 3$, $\vec{U}^{\text{chem}}_{\text{Delta-lig}} \begin{cases} \theta_{\text{Epi-ind}} = 5,\ \theta_{\text{Ant}} = 1 \\ f_{\text{chem}} = \Gamma_{\text{Epi-ind}}\ \text{AND NOT}\ \Gamma_{\text{Ant}} \end{cases}$<br>$\vec{U}^{\text{chem}}_{\text{Delto-lig}} \begin{cases} \theta_{\text{Epi2-ind}} = 5,\ \theta_{\text{Ant}} = 1 \\ f_{\text{chem}} = \Gamma_{\text{Epi2-ind}}\ \text{AND}\ \Gamma_{\text{Ant}} \end{cases}$

Supplementary Table 4 | Parameter values of the epiboly study. See Fig. 5,6. Row titles and equation numbers refer to the corresponding sections and equations. Time step: $\Delta t = 1$ s.

**Mechanical Rules**	$R = 0.030824$ (eq. 1,2), $\lambda = 3000$ (eq. 3), $a = 1.3697$ (eq. 4)<br>$c_{\max} = 1.2414$ (eq. 5), $c_{\text{eq}} = 0.9047$ (eq. 12), $w_{\text{rep}} = w_{\text{adh}} = 4500$ (eq. 14)<br>$T^{\text{cc}} = 1000 \pm 500\Delta t$ (normal law) (eq. 24),<br>$\mu = 0.75$ (eq. 28), $\eta = 0.15$ (eq. 29),
**Protein Degradation** (eq. 36)	Ubi $\begin{cases}\kappa = 0.015,\end{cases}$
**Protein Programming** (eq. 38)	Ubi $\begin{cases}\gamma = 1, \ [t_{\min}, t_{\max}] = [0, +\infty) \\ [x_{\min}, x_{\max}] = [y_{\min}, y_{\max}] = [z_{\min}, z_{\max}] = (-\infty, +\infty)\end{cases}$
**Ligand Degradation** (eq. 42)<br>**Ligand Diffusion** (eq. 44)	Lig $\begin{cases}\chi = 0.02 \\ D = 10\end{cases}$
**Control of Archetype** (eq. 30,31)	Mesenchymal $\begin{cases}\theta_{\text{Ubi}} = 50 \\ f_{\mathcal{M}} = \Gamma_{\text{Ubi}}\end{cases}$
**Control of Polarisation** (eq. 30,31)<br>Chemotactic mode (eq. 52)<br>Bipolarity (eq. 19,20)	$m = 3, \quad \vec{U}^{\text{chem}}_{\text{Lig}} \begin{cases}\theta_{\text{Ubi}} = 5 \\ f_{\text{chem}} = \Gamma_{\text{Ubi}}\end{cases}$
**Control of Protrusion** (eq. 30,31)<br>Active behavioural forces (eq. 57,21)	$\phi = 0.002, \quad \vec{F}^{\text{A}}_{ij} \begin{cases}\theta_{\text{Ubi}} = 5 \\ f_{\text{chem}} = \Gamma_{\text{Ubi}}\end{cases}$

Supplementary Note 1 | Cell state variables

The cell behaviour ontology (CBO) of MecaGen rests on three major "archetypes", which are observed during early embryogenesis across a wide variety of animal embryos: *mesenchymal* cells, denoted by $\mathcal{M}$, *epithelial* cells, denoted by $\mathcal{E}$, and "idle" cells, denoted by $\mathcal{I}$. Epithelial cells can be either polarised ($\mathcal{E}_\pm$) or nonpolarised ($\mathcal{E}_0$). In the formal description of the model, cells are represented by indices $i, j, k, \dots$ and their complete set (the embryo) is denoted by $\mathcal{S} = \mathcal{M} \cup \mathcal{E} \cup \mathcal{I}$, where $\mathcal{E} = \mathcal{E}_\pm \cup \mathcal{E}_0$. We also denote by $\mathcal{S}_0 = \mathcal{S} \setminus \mathcal{E}_\pm = \mathcal{M} \cup \mathcal{E}_0 \cup \mathcal{I}$ the subset of the embryo containing only the nonpolarised cells.

Each cell archetype corresponds to specific biomechanical behaviours. Mesenchymal cells $\mathcal{M}$ are motile entities that can exhibit protrusive activity in any direction determined by their internal polarisation. We assume that there is at most one such direction per cell i, represented by a vector $\vec{U}_i$. In a first stage of the model, $\vec{U}_i$ is assumed given; later, it will be calculated as a function of the cell's molecular and genetic state. Epithelial cells $\mathcal{E}$ support the partitioning of the embryo into morphogenetic fields. Compared to mesenchymal cells, they are also characterised by an intracellular molecular asymmetry that determines an *apicobasal* (AB) polarisation axis, orthogonal to their compartment's border and represented by a vector $\vec{U}_i^{\text{ab}}$. Not every epithelial cell is AB-polarised, however, as this property is context-dependent. Without pressure from other $\mathcal{E}$ neighbours, an isolated cell cannot set up its AB axis, therefore $\vec{U}_i^{\text{ab}} = \vec{0}$ and the cell is categorised in $\mathcal{E}_0$; otherwise, it belongs to $\mathcal{E}_\pm$. Like $\mathcal{M}$ cells, $\mathcal{E}$ cells also possess a basic polarisation vector $\vec{U}_i$, which is either parallel or orthogonal to $\vec{U}_i^{\text{ab}}$ in the case of $\mathcal{E}_\pm$ cells. AB-polarised cells may exhibit *intercalation* in the epithelium plane, via junction remodelling, and *apical constriction* (not included in this version of the model), which pushes lateral neighbours toward the apical domain. Finally, idle cells $\mathcal{I}$ exhibit no active biomechanical behaviour but still offer resistance to deformation.

Each cell i is located in space at coordinates $\vec{X}_i = (x_i, y_i, z_i)$. Its equilibrium shape is represented by an ellipsoidal particle with two characteristic lengths: a principal radius $R_i^\perp$ and a secondary radius $R_i^\parallel$ (twice). In the case of $\mathcal{M}$, $\mathcal{E}_0$, and $\mathcal{I}$ archetypes, these values are equal to a unique radius R_i and the cell is spherical. They differ only for $\mathcal{E}_\pm$ cells, where they can also be denoted by R_i^{ab} and R_i^{lat}, respectively. Accordingly, the volume V_i and surface area A_i of a cell are given by

$$V_i = \begin{cases} \frac{4}{3}\pi R_i^3 & \text{if } i \in \mathcal{S}_0 \\ \frac{4}{3}\pi R_i^\perp R_i^{\parallel 2} & \text{if } i \in \mathcal{E}_\pm, \end{cases} \tag{1}$$

$$A_i = \begin{cases} 4\pi R_i^2 & \text{if } i \in \mathcal{S}_0 \\ 2\pi R_i^{\parallel 2} + \pi R_i^{\perp 2} \left(\frac{1}{\zeta} \ln \frac{1+\zeta}{1-\zeta} \right) & \text{if } i \in \mathcal{E}_\pm \text{ and } R_i^\perp \le R_i^\parallel \\ 2\pi R_i^{\parallel 2} + 2\pi R_i^\perp R_i^\parallel \left(\frac{1}{\zeta'} \arcsin \zeta' \right) & \text{if } i \in \mathcal{E}_\pm \text{ and } R_i^\perp \ge R_i^\parallel, \end{cases} \tag{2}$$

$$\text{where } \zeta = \sqrt{1 - (R_i^\perp / R_i^\parallel)^2} \text{ and } \zeta' = \sqrt{1 - (R_i^\parallel / R_i^\perp)^2}.$$

Inside each cell, the molecular and genetic state is modelled by a list of real-valued quantities $\mathbf{p} = \{p_a\}$ representing the different *proteins* $\{P_a\}$, with $a = 1, \dots, N$, and by binary states $\mathbf{g} = \{g_b\}$ representing the expression levels of *genes* $\{G_b\}$ involved in the gene regulation network (GRN), with $b = 1, \dots, M$. Cell-cell communication is realised via a set of extracellular proteins, called *ligands* $\{Q_c\}$, whose quantities are stored in variables $\mathbf{q} = \{q_c\}$, with $c = 1, \dots, L$.

Supplementary Note 2 | Meca: model of cell biomechanics

The biomechanical model presented here is a type of *particle-based physics*, which means that cells are represented by "particles"—here, only one per cell. The classical Newtonian *equation of motion* describing a particle with mass m and acceleration $\vec{a}$ is given by $m\vec{a} = \vec{F}(\vec{X}, \vec{v}, R^{\parallel}, R^{\perp})$, where the sum of forces $\vec{F}$ generally depends on the particle's location $\vec{X}$, velocity $\vec{v}$, and ellipsoidal radii. However, cells are very small, ambivalent fluid-solid entities[29] submitted to "sticky" interactions, hence their inertial forces are negligible with respect to viscosity (similar to a low Reynolds number in fluid mechanics), modelled by a coefficient λ.[12,13] In this overdamped environment, applied forces become proportional to velocity, not acceleration: with $ma \ll \lambda v$, then $m\vec{a} = -\lambda\vec{v} + \vec{F}$ is equivalent to $\lambda\vec{v} = \vec{F}$.

Generalising to a multicellular system, the motion of each cell i is governed by the sum of forces $\vec{F}_{ij}$ exerted by all other cells j belonging to its neighbourhood $\mathcal{N}_i$:

$$\lambda_i \vec{v}_i = \vec{F}_i = \sum_{j \in \mathcal{N}_i} \vec{F}_{ij} \;=\; \sum_{j \in \mathcal{N}_i} \vec{F}^{\mathrm{P}}_{ij} + \vec{F}^{\mathrm{A}}_{ij} \;=\; \vec{F}^{\mathrm{P}}_{i} + \vec{F}^{\mathrm{A}}_{i}, \tag{3}$$

where $\mathcal{N}_i$ is calculated from cell positions and sizes, $\vec{F}^{\mathrm{P}}_{ij}$ and $\vec{F}^{\mathrm{A}}_{ij}$ are local "passive" and "active" interaction forces exerted by j on i, and viscosity is proportional to the cell's surface area: $\lambda_i = \lambda_0 A_i$. These components are explained in the next sections.

Cell-Cell Contact Area. Although cells are assumed to be ellipsoidal, they are also able to deform in order to interact with farther away cells. If the distance $r_{ij} = \|\vec{X}_i - \vec{X}_j\|$ separating two neighbouring cells i and j is greater than a given maximum distance of deformation $r^{\max}_{ij}$, then the contact area A_{ij} between these two cells vanishes. We propose an empirical law relating A_{ij} to r_{ij}, starting with an approximation of the surface area between two spherical neighbours, then generalising to nonspherical shapes. In the first case, we opt for a simple quadratic law:

$$A_{ij} = A(r_{ij}, R_i, R_j) \approx a(r_{ij} - r^{\max}_{ij})^2 \;\text{ if }\; r_{ij} < r^{\max}_{ij}, \;\text{ otherwise }\; 0, \tag{4}$$

where a is a constant coefficient to be determined. Note that this is different from the intersection of two spheres, which forms a lens whose dimensions are known exactly. The above formula represents the contact area obtained by *pressing* two "soft" spheres against each other. The choice of degree 2 is motivated by physical units and scaling considerations, since A should clearly verify: $A(2r_{ij}, 2R_i, 2R_j) = 4A(r_{ij}, R_i, R_j)$. This also leads us to assume that $r^{\max}_{ij}$ is roughly proportional to the cells' radii:

$$r^{\max}_{ij} \approx c_{\max}(R_i + R_j), \tag{5}$$

where $c_{\max}$ is another empirical coefficient. In the absence of real data, we constructed an artificial testbed experiment to infer the values of a and $c_{\max}$. It consisted of an ellipsoidal domain filled with three consecutive generations of dividing cells, then distorted in various ways to force the cells into different spatial rearrangements. We found the best fit to occur for $a = 1.3697$ and $c_{\max} = 1.2414$, and used these values for all pairs of spherical cells (i.e. belonging to $\mathcal{S}_0$) throughout the model.

In the case of a pair of neighbours comprising a spherical cell $i \in \mathcal{S}_0$ and an nonspherical cell $j \in \mathcal{E}_\pm$, the relation remains the same provided which radius R_j is used. To this goal, we must establish whether the spherical cell i lies in the AB or lateral spatial domain of the nonspherical cell j, denoted by $\mathcal{D}^{\mathrm{ab}}_j$ and $\mathcal{D}^{\mathrm{lat}}_j$ respectively (Supplementary Fig. 1). This is done by comparing the scalar product of $\vec{u}_{ij}$, the unit edge vector from j to i, and $\vec{U}^{\mathrm{ab}}_j$, the AB polarisation vector of the epithelial cell, to a threshold β_j (the cosine of half a cone's aperture):

$$\begin{cases} i \in \mathcal{D}^{\mathrm{ab}}_j \;\Leftrightarrow\; |\vec{u}_{ij} \cdot \vec{U}^{\mathrm{ab}}_j| \geq \beta_j \\ i \in \mathcal{D}^{\mathrm{lat}}_j \;\Leftrightarrow\; |\vec{u}_{ij} \cdot \vec{U}^{\mathrm{ab}}_j| < \beta_j, \end{cases}$$

$$\text{where } \vec{u}_{ij} = \frac{\vec{X}_j - \vec{X}_i}{\|\vec{X}_j - \vec{X}_i\|}, \text{ and } \beta_j = \frac{R_j^{\perp}}{\sqrt{R_j^{\perp 2} + R_j^{\| 2}}}. \tag{6}$$

If i lies in the AB domain of j, then $R_j^{\text{ab}} = R_j^{\perp}$ is used and $A_{ij} = A(r_{ij}, R_i, R_j^{\perp})$ with $r_{ij}^{\max} = c_{\max}(R_i + R_j^{\perp})$. If i lies in the lateral domain of j, then it is $R_j^{\text{lat}} = R_j^{\|}$ and $A_{ij} = A(r_{ij}, R_i, R_j^{\|})$ with $r_{ij}^{\max} = c_{\max}(R_i + R_j^{\|})$.

Finally, when two nonspherical cells touch, each cell verifies to which one of its neighbour's domain it belongs, and the contact area is determined accordingly from four possible combinations: for example, $A_{ij} = A(r_{ij}, R_i^{\perp}, R_j^{\perp})$ with $r_{ij}^{\max} = c_{\max}(R_i^{\perp} + R_j^{\perp})$. Note that the total cell surface area A_i of cell i cannot be calculated by adding all the known contact areas A_{ij} because some cells are not completely surrounded by neighbours in contact with them. Therefore A_i is still approximated by the spherical or ellipsoidal formulas seen above.

Cell Neighbourhood. The neighbourhood $\mathcal{N}_i$ of cell i is calculated in two steps: first, a preselection of potential neighbours that obey certain *metric* criteria, then a refinement of this list according to *topological* features. Two cells i, j at locations $\vec{X}_i$, $\vec{X}_j$ are considered to be metric neighbours simply if their distance $r_{ij} = \|\vec{X}_i - \vec{X}_j\|$ is smaller than a constant cut-off value:

$$\mathcal{N}_i^{\text{m}} = \left\{ j \in \mathcal{S} : r_{ij} \le r_{ij}^{\max} \right\}. \tag{7}$$

This purely metric neighbourhood, however, is not viable as it often leads to volumes collapsing during simulation when the adhesion strength between interacting cells is high. This is why we opt for a topological neighbourhood, denoted by $\mathcal{N}_i$, based on a variant of the Voronoi diagram and its dual, the Delaunay triangulation, called a *Gabriel graph*. In 2D, this method imposes that no node be found inside the circle whose diameter is a valid neighbourhood edge. We generalise the Gabriel criterion to the 3D case using spheres (instead of triangular simplexes) as follows:

$$\mathcal{N}_i = \left\{ j \in \mathcal{N}_i^{\text{m}} : \forall k \in \mathcal{N}_i^{\text{m}}, \|\vec{X}_k - \frac{1}{2}(\vec{X}_i + \vec{X}_j)\| \ge \frac{r_{ij}}{2} \right\}. \tag{8}$$

In the case of epithelial cells, we distinguish between the neighbours of i located in its AB domain and those located in its lateral spatial domain: $\mathcal{N}_i^{\text{ab}} = \mathcal{N}_i \cap \mathcal{D}_i^{\text{ab}} = \{ j \in \mathcal{N}_i : |\vec{u}_{ij} \cdot \vec{U}_i^{\text{ab}}| \ge \beta_i \}$ and $\mathcal{N}_i^{\text{lat}} = \mathcal{N}_i \cap \mathcal{D}_i^{\text{lat}} = \{ j \in \mathcal{N}_i : |\vec{u}_{ij} \cdot \vec{U}_i^{\text{ab}}| < \beta_i \}$, respectively.

Apicobasal Axis in Epithelial Cells. We explain here the method used to calculate the AB polarisation axis of an epithelial cell. Our underlying hypothesis is that AB polarity can be established or maintained only if the considered $\mathcal{E}$ cell is surrounded by other $\mathcal{E}$ cells in its lateral neighbourhood (whether polarised or not). An epithelial cell that is isolated or only surrounded by mesenchymal cells is not able to polarise.

The AB axis is obtained via an update procedure based on the topological neighbourhood. The surface orientation of the epithelium at each cell i is set to the average of the outward normal vectors of the n triangles formed by the n epithelial neighbours located in the lateral domain of the cell (Supplementary Fig. 2). These triangles are obtained by sorting the list of neighbours anticlockwise around the current axis $\vec{U}_i^{\text{ab}}$. Denoting by a_k the neighbouring indices in the sorted list, with $k = 0, ..., n-1$, and defining an extra index $a_n = a_0$ for convenience, the n surrounding triangles are given by $\widehat{X_{a_k} X_i X_{a_{k+1}}}$. The updated cell axis is then calculated by summing cross products and normalising:

$$\vec{U}_i^{\text{ab}} = \frac{1}{n} \sum_{i=0}^{n-1} \frac{(\vec{X}_{a_k} - \vec{X}_i) \times (\vec{X}_{a_{k+1}} - \vec{X}_i)}{\|(\vec{X}_{a_k} - \vec{X}_i) \times (\vec{X}_{a_{k+1}} - \vec{X}_i)\|}, \quad \text{then } \vec{U}_i^{\text{ab}} \leftarrow \frac{\vec{U}_i^{\text{ab}}}{\|\vec{U}_i^{\text{ab}}\|}. \tag{9}$$

Note that cells at the border of an epithelial domain do not have neighbours all around them. In that case, the pseudo-triangle formed with its two neighbours along the same border should not be taken into account. This is done by removing any triangle $\widehat{X_{a_k} X_i X_{a_{k+1}}}$ whose angle is greater than π, i.e. whose cross-product is pointing inward.

A particular situation occurs when the epithelial cell is not yet polarised (or "pre-epithelial"). Since such a cell does not possess an AB axis, another vector is used in lieu of $\vec{U}_i^{\text{ab}}$ to calculate the sorted list of neighbours. The polarisation axis that this potential vector represents will be regulated by the cell's genetic and molecular state (see "Control" sections toward the end).

Cell-Cell Forces. The total force $\vec{F}_{ij}$ exerted by j over i is the sum of two components (equation 3):

- a passive *relaxation* force $\vec{F}_{ij}^{\text{P}}$, which maintains the integrity of the cell's volume and controls both the stiffness and the adhesion of the interaction. This force is always applied irrespective of the state of the cell, but can be modulated. In the case of an epithelial cell j, we add an extra term representing the force exerted on its lateral neighbours to allow for conservation of graph planarity and control the rigidity of the epithelium.
- an active *behavioural* force $\vec{F}_{ij}^{\text{A}}$, which gives rise to specific behaviours depending on the molecular state of cell i. All differentiated cells, whether mesenchymal or epithelial, exhibit an active behaviour: $\mathcal{M}$ cells can exert a protrusive activity leading to an intercalation behaviour, while $\mathcal{E}$ cells can also intercalate in the tangential plane of the epithelium via junction remodelling, and bend the local curvature via apical constriction (not included in this version of the model).

Passive Relaxation Forces. The passive relaxation force $\vec{F}_{ij}^{\text{P}}$ exerted by a cell j over a cell i is itself the sum of two components:

$$\vec{F}_{ij} = (\vec{F}_{ij}^{\text{P,ar}} + \vec{F}_{ij}^{\text{P,pc}}) + \vec{F}_{ij}^{\text{A}}, \tag{10}$$

- an *attraction-repulsion* interaction force $\vec{F}_{ij}^{\text{P,ar}}$, which maintains the integrity of the cell's volume and controls the interaction via a stiffness coefficient and an adhesion coefficient
- a *planarity conservation* interaction force $\vec{F}_{ij}^{\text{P,pc}}$, which maintains the planarity of a monolayered epithelium via a rigidity coefficient.

Taken together, passive forces lead the swarm of cells toward an equilibrium state characterised by optimal distances between centres.

Passive Relaxation Forces: Attraction-Repulsion. We introduce here the general expression of the passive attraction-repulsion (AR) force $\vec{F}_{ij}^{\text{P,ar}}$ between two neighbouring spherical cells i and j with radii R_i and R_j. If i is nonspherical, R_i must be set to one of $R_i^{\perp}$ or $R_i^{\parallel}$ depending on whether j belongs to the AB neighbourhood $\mathcal{N}_i^{\text{ab}}$ or lateral neighbourhood $\mathcal{N}_i^{\text{lat}}$ of i, respectively. If both cells are nonspherical, then the same applies to R_j.

The AR force is parallel to the edge vector $\vec{u}_{ij}$, and its magnitude is the derivative of a relaxation potential $E_{ij}^{\text{P,ar}}$ composed of three distance intervals:

- a *repulsion* interval (sharply decreasing E) for distances shorter than an equilibrium distance defined by $r_{ij}^{\text{eq}} = c_i^{\text{eq}} R_i + c_j^{\text{eq}} R_j$, where in the most general case c_i^{eq} is a coefficient that varies from cell to cell
- an *attraction* interval (slowly increasing E) for distances over r_{ij}^{eq}
- a *neutral* interval (constant E) beyond the maximum limit of the interaction field $r_{ij}^{\max} = c_{\max}(R_i + R_j)$.

The rationale of E is to maximise the contact area between neighbouring cells. To calculate the equilibrium coefficient c_i^{eq}, we consider that the equilibrium state of the swarm is reached when the cells form the densest packing in the 2D plane, i.e. a hexagonal lattice (Supplementary Fig. 3). In this perfectly uniform arrangement, the ratio of the total surface area occupied by the cells is equal to the ratio of one disk to one hexagon, which is $\pi/(2\sqrt{3}) \simeq 0.9069$. Hence, the equilibrium distance between two neighbouring cells (i, j) of common radius R is constant and reads

$$r_{\text{eq}} = 2c_{\text{eq}}R, \text{ with } c_{\text{eq}} = \sqrt{\frac{\pi}{2\sqrt{3}}} \simeq 0.9523. \tag{11}$$

In the case of unequal radii R_i and R_j, c_i^{eq} is approximated by c_{eq} and the equilibrium distance becomes the following linear combination:

$$r_{ij}^{\text{eq}} = c_{\text{eq}}R_i + c_{\text{eq}}R_j = c_{\text{eq}}(R_i + R_j). \tag{12}$$

Under these conditions, we consider here the simplest form of schematic mechanical model that can realise both the short-range repulsion and long-range attraction parts, namely *spring-like forces derived from an elastic potential*. It means that potential $E_{ij}^{\text{P,ar}}(r)$ is a quadratic function of $r - r_{\text{eq}}$, and $\vec{F}_{ij}^{\text{P,ar}}(r)$ is a linear function. Although mechanical interactions between neighbouring cells have been extensively studied in biophysics, the multiprotein and polymeric nature of the cytoskeleton added to the membrane's "fluid mosaic" flexibility and adhesiveness make it a very elusive object, which is difficult to summarise into a definitive set of equations. Roughly, the consensus hypothesis is that a major driving mechanism can be characterised by intercellular *surface tension*. The two main components of this tension are cellular *adhesion*, which decreases it, and cellular *cortical tension*, which acts antagonistically. The balance between these two components determines the ultimate behaviour of the interaction, i.e. whether it is attractive or repulsive.

We take into account another fundamental principle, *cell volume conservation*, in the repulsive part of the potential. Accordingly, the intensity of "adhesion" (which refers to the combined effect of true surface adhesion and its antagonist, cortical tension) is modulated independently from the intensity of repulsion by using two different stiffness coefficients, w_{adh} and w_{rep}, respectively above and below the equilibrium distance (Supplementary Fig. 4):

$$\vec{F}_{ij}^{\text{P,ar,lin}} = \begin{cases} -w_{\text{rep}}(r_{ij} - r_{ij}^{\text{eq}})\,\vec{u}_{ij} & \text{if } r_{ij} < r_{ij}^{\text{eq}} \\ -w_{\text{adh}}(r_{ij} - r_{ij}^{\text{eq}})\,\vec{u}_{ij} & \text{if } r_{ij}^{\text{eq}} \leq r_{ij} < r_{ij}^{\text{max}}. \\ \vec{0} & \text{if } r_{ij}^{\text{max}} \leq r_{ij} \end{cases} \tag{13}$$

This, however, is only the linear component of $\vec{F}^{\text{P,ar}}$ because the intensity of the attraction cannot be suddenly maximal in its most distant point r_{ij}^{max}, coming from the right. For a more realistic interaction model, we multiply the linear force $\vec{F}_{ij}^{\text{P,ar,lin}}$ by the contact area $A(r_{ij}, R_i, R_j)$ (a half-parabola shape for $r < r_{ij}^{\text{max}}$) so that it vanishes when i and j are no longer touching:

$$\begin{aligned} \vec{F}_{ij}^{\text{P,ar}} &= A_{ij}\vec{F}_{ij}^{\text{P,ar,lin}} \\ &= \begin{cases} -w_{\text{rep}}\,a(r_{ij} - r_{ij}^{\text{max}})^2(r_{ij} - r_{ij}^{\text{eq}})\,\vec{u}_{ij} & \text{if } r_{ij} < r_{ij}^{\text{eq}} \\ -w_{\text{adh}}\,a(r_{ij} - r_{ij}^{\text{max}})^2(r_{ij} - r_{ij}^{\text{eq}})\,\vec{u}_{ij} & \text{if } r_{ij}^{\text{eq}} \leq r_{ij} < r_{ij}^{\text{max}}. \\ \vec{0} & \text{if } r_{ij}^{\text{max}} \leq r_{ij} \end{cases} \end{aligned} \tag{14}$$

A comparison of our custom attraction-repulsion force $\vec{F}_{ij}^{\text{P,ar}}$ with classical potentials such as Morse and Lennard-Jones is shown in Supplementary Fig. 5.

Passive Relaxation Forces: Planarity Conservation. Attraction-repulsion forces, which are parallel to the neighbourhood edges, are not sufficient to keep the layout of an epithelium planar in 3D space. If left alone, these forces would eventually make the epithelium condense into a multi-layered tissue or a clump. To avoid this problem, we introduce *planarity conservation* (PC) forces specific to a monolayered epithelial sheet, denoted by $\vec{F}_{ij}^{\text{P,pc}}$ (Supplementary Fig. 6). Their purpose is to account for the fact that each neighbour i is maintained in the lateral neighbourhood $\mathcal{N}_j^{\text{lat}}$ of j, close to the tangential plane of the epithelium. A force $\vec{F}_{ij}^{\text{P,pc}}$ exerted by j over i is parallel to the normalised sum of both AB polarisation vectors, which we call "edge-AB vector" and denote by $\vec{n}_{ij}$ (as a lighter alternative to $\vec{u}_{ij}^{ab}$):

$$\vec{n}_{ij} = \frac{\vec{U}_i^{\text{ab}} + \vec{U}_j^{\text{ab}}}{\|\vec{U}_i^{\text{ab}} + \vec{U}_j^{\text{ab}}\|}. \tag{15}$$

The magnitude of $\vec{F}_{ij}^{\text{P,pc}}$ is the product of four contributions:

- a rigidity coefficient k_{rig} controlling the planarity of the epithelium
- the dot product between the edge vector $\vec{u}_{ij}$ and the edge-AB vector $\vec{n}_{ij}$, representing the fact that $\vec{F}_{ij}^{\text{P,pc}}$ must be zero when the epithelium is perfectly planar, i.e. when the AB polarisation vectors are perpendicular to the edge vectors
- a "cell shape factor", $R_i^{\text{ab}}/R_i^{\text{lat}} = R_i^{\perp}/R_i^{\parallel}$, motivated by the fact that columnar epithelium cells, which are taller than wide, are more effective at conserving planarity than squamous cells, which are wider than tall
- a surface scaling term proportional to ${R_i^{\text{ab}}}^2$.

Taking the opposite viewpoint, cell i exerts the same force on a cell j located in its lateral neighbourhood $\mathcal{N}_i^{\text{lat}}$. Conversely, however, i may not belong to $\mathcal{N}_j^{\text{lat}}$. For this reason, we distinguish two types of planarity conservation forces: "intrinsic" and "extrinsic". In this schema, the force generated by i induces a torque transmission from i to j, with $\vec{F}_{ij}^{\text{P,pc,int}}$ being the component responsible for the motion of i and $\vec{F}_{ji}^{\text{P,pc,ext}}$ its counterpart, responsible for the motion of j, which have same magnitude but opposite directions: $\vec{F}_{ij}^{\text{P,pc,int}} = -\vec{F}_{ji}^{\text{P,pc,ext}}$. This modelling technique is explained in more detail in the next section, where behavioural forces are exerted by an "active" cell on a neighbouring cell that may not be itself active. Altogether, the PC forces mutually exerted between two neighbouring epithelial cells i and $j \in \mathcal{N}_i^{\text{lat}}$ are

$$\vec{F}_{ij}^{\text{P,pc,int}} = k_{\text{rig}}({R_i^{\text{ab}}}^3/R_i^{\text{lat}})(\vec{u}_{ij}\cdot\vec{n}_{ij})\,\vec{n}_{ij} \quad\text{and}\quad \vec{F}_{ji}^{\text{P,pc,ext}} = -\vec{F}_{ij}^{\text{P,pc,int}}. \tag{16}$$

Therefore, noting that $\vec{u}_{ji} = -\vec{u}_{ij}$ and $\vec{n}_{ji} = \vec{n}_{ij}$, the total PC force exerted on i reads

$$\begin{aligned}\vec{F}_i^{\text{P,pc}} &= k_{\text{rig}} \sum_{j\in\mathcal{N}_i^{\text{lat}}} \left(\vec{F}_{ij}^{\text{P,pc,int}} + \vec{F}_{ij}^{\text{P,pc,ext}}\right)\\ &= k_{\text{rig}} \sum_{j\in\mathcal{N}_i^{\text{lat}}} \left(\frac{{R_i^{\text{ab}}}^3}{R_i^{\text{lat}}} + \frac{{R_j^{\text{ab}}}^3}{R_j^{\text{lat}}}\right)(\vec{u}_{ij}\cdot\vec{n}_{ij})\,\vec{n}_{ij}.\end{aligned} \tag{17}$$

Active Behavioural Forces. Another crucial difference with solid objects is that surrounding cells are responsible not only for damping motion but also for motion itself. To progress, a cell needs to "cling on" and "push back" surrounding cells, somewhat like a swimmer needs to push back water to move forward. To

reflect this, we also introduce active behavioural forces, denoted by $\vec{F}_{ij}^{\mathrm{A}}$, composed of an *intrinsic* term $\vec{F}_{ij}^{\mathrm{A,int}}$ and its *extrinsic* counterpart $\vec{F}_{ij}^{\mathrm{A,ext}}$, whose purpose is to represent a schematic model of the cells' specialised biomechanics:

$$\vec{F}_{ij} = (\vec{F}_{ij}^{\mathrm{P,ar}} + \vec{F}_{ij}^{\mathrm{P,pc}}) + (\vec{F}_{ij}^{\mathrm{A,int}} + \vec{F}_{ij}^{\mathrm{A,ext}}). \tag{18}$$

Active Behavioural Forces: Mesenchymal Protrusion. Cell protrusion, most apparent in $\mathcal{M}$ cells, is essentially based on a *treadmilling* activity somewhat similar to tracked vehicles (such as tanks) except that, since inertia plays no part in cellular interactions, adhesion is regulated in a special way to avoid sliding between the cell surfaces in contact. Protrusive activity induces an *intercalation* of the cell between its neighbours. One condition is the presence of a polarisation axis $\vec{U}_i$ (different from $\vec{U}_i^{\mathrm{ab}}$, which $\mathcal{M}$ cells do not possess), generally created by the diffusion of extracellular ligand molecules and an asymmetrical distribution of intracellular substances. This axis can also be due to mechanotransduction from neighbouring cell-cell contacts, or feedback from the active forces themselves, but these aspects are not modelled here. In any case, $\vec{U}_i$ determines two regions of the cell, or *poles*, where protrusive activity occurs: if only one pole is active, the cell is called "monopolar"; if both are active, it is called "bipolar".

At the subcellular level, the main structure underlying protrusion is the *cytoskeleton*. We focus here on the cell cortex, a mesh-like network made of actin and myosin molecules that lie just below the plasma membrane and are attached to it by molecular complexes including catenins. The active deformation of this actomyosin network, essentially by actin polymerisation, provides the driving mechanism of protrusion (Supplementary Fig. 7). At the molecular level, focal adhesion points are made and unmade between neighbouring cells, while monomers assemble into actin at the plus end, and disassemble at the minus end.

The coupled action of the actomyosin cortex and the focal adhesion points forms a sort of "treadmill" originating at the tip of the protrusion (Supplementary Fig. 8). The contrary movement of the protruding tip and the nearest membranes tied to it via focal adhesion points is analogous to torque transmission between two large adjacent discs via tiny ball bearings. The transfer of cell material in the bulge results from the "intrinsic" force generated by the actomyosin cortex, while the "extrinsic" force is exerted on the adjacent cell through the focal adhesion points. In some cases, an additional mechanism of cellular contraction at the back of the cell amplifies the intrinsic force (not included here). We assume that the distribution of focal adhesion points is homogeneous on the surface area of the cell, so the quantity of torque transmitted between two neighbouring cells i and j is proportional to their contact area A_{ij}.

In our particle-based framework, the mathematical interpretation and representation of this mechanism is the following. Among the neighbouring cells in $\mathcal{N}_i$, we denote by $\mathcal{N}_i^+$ the subset of neighbours that make contact with cell i on its "positive" pole, i.e. which are positioned relative to i in the same general direction as its polarisation axis $\vec{U}_i$ (Supplementary Fig. 9A, green pie-slice domain covering two cells j and k):

$$\mathcal{N}_i^+ = \left\{ j \in \mathcal{N}_i : \vec{u}_{ij} \cdot \vec{U}_i \geq \eta \right\}, \tag{19}$$

a definition similar to, but not to be confused with, the AB neighbourhood $\mathcal{N}_i^{\mathrm{ab}} = \{ j \in \mathcal{N}_i : |\vec{u}_{ij} \cdot \vec{U}_i^{\mathrm{ab}}| \geq \beta_i \}$ of an $\mathcal{E}$ cell. A constant threshold value η controls here the relative size of the protrusion (the opening of the pie slice). Similarly, we denote by $\mathcal{N}_i^-$ the subset of neighbours that share a contact area on the "negative" pole of the cell, i.e. away from the polarisation vector:

$$\mathcal{N}_i^- = \left\{ j \in \mathcal{N}_i : \vec{u}_{ij} \cdot \vec{U}_i \leq -\eta \right\}. \tag{20}$$

This neighbourhood is used in the case of opposite monopolar, or bipolar, protrusion.

In the regular monopolar case illustrated here, for each neighbour $j \in \mathcal{N}_i^+$, a pair of equal and opposite forces contribute to the motion of both i and j: an intrinsic force $\vec{F}_{ij}^{\mathrm{A,int}}$ (larger dashed green arrow) and

its simultaneous and exact extrinsic counterpart $\vec{F}_{ji}^{\text{A,ext}} = -\vec{F}_{ij}^{\text{A,int}}$ (larger dashed red arrow). The common axis of these forces is designed to roughly emulate the profile of the contact area that can be seen in the polygonal representation of Supplementary Fig. 8 but is not shown in the disc-particle representation of Supplementary Fig. 9. It is a linear combination of the polarisation axis $\vec{U}_i$ and its orthogonal complement $\vec{U}_i^{\perp j}$ passing through j:

$$\vec{F}_{ij}^{\text{A,int}} = \varphi_{\text{prot}} A_{ij} \left(\cos\nu\, \vec{U}_i + \sin\nu\, \vec{U}_i^{\perp j} \right) \text{ and } \vec{F}_{ji}^{\text{A,ext}} = -\vec{F}_{ij}^{\text{A,int}},$$

$$\text{where } \vec{U}_i^{\perp j} = \frac{\vec{u}_{ij} - (\vec{u}_{ij} \cdot \vec{U}_i)\, \vec{U}_i}{\|\vec{u}_{ij} - (\vec{u}_{ij} \cdot \vec{U}_i)\, \vec{U}_i\|}, \tag{21}$$

the angle ν tunes the profile of the contact area (small dashed arrows resulting in the larger dashed arrows), and the protrusion coefficient φ_{prot} tunes the intensity of the force. We could precisely calculate ν as a function of the angular position of each neighbour, but this sophistication is unnecessary. We chose instead a constant value of $\arctan(4/3) \approx 53°$ (experiments with other values around 45° showed no significant difference).

Suppose for now that only cell i exerts a monopolar protrusive activity on its neighbours (Supplementary Fig. 9B). For each $j \in \mathcal{N}_i^+$, this creates a pair of forces $\vec{F}_{ij}^{\text{A,int}}$ and $\vec{F}_{ji}^{\text{A,ext}}$ as described above. Therefore, the total behavioural force generated by i's protrusion (solid green arrow, not necessarily parallel to $\vec{U}_i$ in 3D) is the sum of its neighbours' contributions: $\vec{F}_i^{\text{A,int}} = \vec{F}_{ij}^{\text{A,int}} + \vec{F}_{ik}^{\text{A,int}}$ (dashed green arrows). Symmetrically, if j also protrudes, then an equivalent set of forces is created around it (Supplementary Fig. 9C). In that case, combining both protruding activities from i and j, each cell in the neighbourhood can be the site of both intrinsic and extrinsic forces (Supplementary Fig. 9D): the former come from its own protruding activity (green arrows), the latter from the protruding activity of its neighbours (red arrows). In this particular illustration, the third cell k is not protruding, thus its own total active force is only made of extrinsic components coming from i and j. The sum of 'int' and 'ext' forces on i (resp. j and k) yields the net "active" force on this cell (not shown), which corresponds to $\vec{F}_i^{\text{A}}$ (resp. $\vec{F}_j^{\text{A}}$ and $\vec{F}_k^{\text{A}}$) in its motion equation. Each net active force leads a cell to move alongside its neighbours and pass through.

At the scale of the whole embryo, the net global force resulting from this complex field of local intrinsic/extrinsic active forces should be zero, due to their mutual compensation:

$$\sum_i \sum_{j \in \mathcal{N}_i} \vec{F}_{ij}^{\text{A}} = \sum_i \vec{F}_i^{\text{A}} = \vec{0}. \tag{22}$$

This is a reasonable requirement, as the relative movements of cells with respect to each other (protrusion, migration, constriction, etc.) should not have the effect of moving the embryo but only reshaping it.

Active Behavioural Forces: Epithelial junction remodelling. Epithelial cells are also able to intercalate themselves between other epithelial cells in a manner similar to mesenchymal cells. In MecaGen, the only difference in this behaviour of $\mathcal{E}$ cells with respect to the protrusive behaviour of $\mathcal{M}$ cells is the fact that this intercalation behaviour is confined to the plane of the epithelium. To reflect this property, the relative neighbourhood vector $\vec{u}_{ij}$ is replaced here by its projection $\vec{u}'_{ij}$ on the plane orthogonal to $\vec{U}_i^{\text{ab}}$, and the expression of the orthogonal complement becomes

$$\vec{U}_i'^{\perp j} = \frac{\vec{u}'_{ij} - (\vec{u}'_{ij} \cdot \vec{U}_i)\, \vec{U}_i}{\|\vec{u}'_{ij} - (\vec{u}'_{ij} \cdot \vec{U}_i)\, \vec{U}_i\|}, \text{ where } \vec{u}'_{ij} = \vec{u}_{ij} - (\vec{u}_{ij} \cdot \vec{U}_i^{\text{ab}})\, \vec{U}_i^{\text{ab}}, \tag{23}$$

which replaces $\vec{U}_i^{\perp j}$ in equation (21).

Cell Cycle and Mitosis. The division of cell i into two cells, or "mitotis", is triggered recursively every time a *cell cycle* is completed, i.e. when the amount of time elapsed since the last mitosis is equal to a (randomly generated) cell cycle length T_i^{cc}. In MecaGen, two phases are distinguished within one cycle: a constant time interval T_0 before cytokinesis (constriction of the membrane and separation of the cells), during which the cell does not display any active behaviour, and a variable time interval T_i^{ac} after cytokinesis, during which the cell can be active; thus $T_i^{\mathrm{cc}} = T_0 + T_i^{\mathrm{ac}}$. The total cell cycle length may vary during the time frame of embryonic development, depending on the type of the cell and its position in the organism or the morphogenetic field it belongs to. We do not assume here specific interactions between the molecular state of the cell and the evolution of its cycle length. Instead, we propose a simple lineage law, in which daugther cell's cycle length T_d^{cc} is a direct function of its mother's cycle length T_i^{cc}, via either a geometric or an arithmetic progession rule:

$$T_d^{\mathrm{cc}} = \min(T_0, \widehat{T_i^{\mathrm{cc}}}) \text{ with } \widehat{T} = r_d T \text{ or } \widehat{T} = t_d + T, \tag{24}$$

where the rate r_d is uniformly distributed in an interval around r_0, and the increment t_d similarly around t_0. If these two simple rules are not sufficient to cover a more diverse array of cell cycle evolution during development, then customised equations can be added to the MecaGen framework.

Beside the temporal dimension, the *spatial coordinates* of daughter cells must also be calculated. First, a mitosis axis $\vec{M}_i$ is chosen in accordance with the mother cell type:

- In nonpolarised (spherical) cells, $\vec{M}_i$ is isotropic:

$$\forall i \in \mathcal{S}_0,\ \vec{M}_i = x_1\vec{e}_1 + x_2\vec{e}_2 + x_3\vec{e}_3, \text{ followed by } \vec{M}_i \leftarrow \frac{\vec{M}_i}{\|\vec{M}_i\|}, \tag{25}$$

  where $x_{1,2,3}$ are random coordinates uniformly drawn in the interval $[-1,1]$ (yielding an approximate uniform distribution after normalisation on the unit sphere) and $\vec{e}_{1,2,3}$ are orthonormal vectors.

- In AB-polarised epithelial cells, $\vec{M}_i$ belongs to the local tangential plane of the epithelium. This plane is defined by a pair of orthonormal vectors $(\vec{U}_i^{\mathrm{ep}}, \vec{U}_i'^{\mathrm{ep}})$ inferred from the axis $\vec{U}_i^{\mathrm{ab}} = (u_1, u_2, u_3)$ as follows:

$$\vec{U}_i^{\mathrm{ep}} = \frac{1}{\sqrt{u_1^2 + u_2^2}}(-u_2, u_1, 0) \text{ and } \vec{U}_i'^{\mathrm{ep}} = \vec{U}_i^{\mathrm{ab}} \times \vec{U}_i^{\mathrm{ep}}, \tag{26}$$

  where $\vec{U}_i^{\mathrm{ep}}$ is an arbitrary vector perpendicular to $\vec{U}_i^{\mathrm{ab}}$, chosen in the $(\vec{e}_1, \vec{e}_2)$ plane for convenience, and $\vec{U}_i'^{\mathrm{ep}}$ completes that 3D basis in the sought-after tangential plane. With this, the division axis becomes

$$\forall i \in \mathcal{E}_\pm,\ \vec{M}_i = x_1\vec{U}_i^{\mathrm{ep}} + x_2\vec{U}_i'^{\mathrm{ep}}, \text{ followed by } \vec{M}_i \leftarrow \frac{\vec{M}_i}{\|\vec{M}_i\|}, \tag{27}$$

  where $x_{1,2}$ are random coordinates uniformly drawn in the interval $[-1,1]$ (yielding an approximate uniform distribution after projection on the unit circle).

Then, the geometry of the daughter cells is determined by assuming that division is symmetrical, i.e. both cells have the same volume. It means that the daughters' dimensions are identical and inferred from the mother's as follows:

$$V_i = \begin{cases} \forall i \in \mathcal{S}_0,\ R_d = \sqrt[3]{\mu} R_i \\ \forall i \in \mathcal{E}_\pm,\ R_d^{\|} = \sqrt{\mu} R_i^{\|} \text{ and } R_d^{\perp} = R_i^{\perp}, \end{cases} \tag{28}$$

where μ is a volume decrease factor such that $V_d = \mu V_i$, with $\mu = 0.5$ for "strict" divisions involving conservation of total volume (when a large mother cell divides into two half-size daughter cells), and

$0.5 < \mu \leq 1$ when division is accompanied by cell growth. For epithelial cells, the AB axes are also identical, i.e. $\vec{U}_d^{\mathrm{ab}} = \vec{U}_i^{\mathrm{ab}}$. Finally, the two daughter cells are positioned on opposite sides of the (disappeared) mother along its division axis $\vec{M}_i$, and at a distance proportional to its radius:

$$\begin{cases} \vec{X}_{d_1} = \vec{X}_i + \eta R_i \vec{M}_i \\ \vec{X}_{d_2} = \vec{X}_i - \eta R_i \vec{M}_i, \end{cases} \tag{29}$$

where η is a scaling factor typically equal to 0.15.

Supplementary Note 3 | Gen: Model of genetic regulation and molecular signalling

The next sections review the principles underlying the cells' chemical activity. The Gen part of the model is based on three simple rule sets that can be easily computed: rules driving the dynamics of intracellular gene and protein reactions, rules driving the dynamics of ligand secretion (out of the cell) and messenger transduction (into the cell) by ligand-receptor binding, which link the intracellular with the extracellular milieu, and rules driving the dynamics of extracellular ligand transport and diffusion.

Following a *chemical kinetic* framework, protein rules are represented in their most general form by ordinary differential equations (ODEs) of the type $d\mathbf{p}/dt = f(\mathbf{p}, \mathbf{g}, \mathbf{q}, \boldsymbol{\rho})$, where $\mathbf{p}$ represents a list of protein concentrations, $\mathbf{g}$ gene expression levels, $\mathbf{q}$ extracellular ligand concentrations, and $\boldsymbol{\rho}$ membrane receptor concentrations. Taking into account spatial diffusion, too, ligand rules obey partial differential equations (PDEs) in $\partial\mathbf{q}/\partial t$.

Genes and Proteins. Variables $\mathbf{p} = \{p_a\}$, with $a = 1, ..., N$, are real-valued quantities representing the concentrations of different protein types $\{P_a\}$, i.e. in each case the number of molecules divided by the volume of the cell. Variables $\mathbf{g} = \{g_b\}$, with $b = 1, ..., M$, represent the expression levels of genes $\{G_b\}$ involved in the GRN, and are restricted here to binary values: $g_b \in [0, 1]$. When a cell divides, we assume that its various protein concentrations are inherited by the two daughter cells without discontinuities.

Bypassing the role of RNA in this model, transcription and translation are construed as a single process, thus the term "protein" implicitly includes mRNA. A protein can be involved in a protein-protein reaction, or act as a transcription factor binding a gene regulatory site. We also assume that each gene G_b produces a single protein type $P_{a=b}$, although the cell can contain more protein types than genes, i.e. $N \geq M$. This is because the GRN model only covers a small part of the whole genome, while other proteins can originate from regulatory circuits not simulated here or from other mechanisms. Proteins synthesized by identified genes are indexed in the same order: $a = b \in [1, M]$, then proteins of other origins are indexed in the interval $[M+1, N]$ if it exists.

The following sections describe the dynamics of genes and proteins. Gene activities $\mathbf{g}$ are regulated by proteins via Boolean functions representing a logical combination of activators and repressors. Protein concentrations $\mathbf{p}$ evolve according to three rules: de novo synthesis by identified genes, degradation, and protein-protein interactions. Additionally, they can also be "preprogrammed" as input in certain spatiotemporal domains of the embryo.

Gene Expression. The activity of a gene G_b is enhanced by the "presence" of certain *activating* transcription factors (TFs, subsumed under the term "protein" here) and/or the "absence" of certain *repressing* TFs. Both types of TFs bind to the regulatory sites of the gene, which are regions of the DNA controlling protein expression. We denote by $P_a \curvearrowright G_b$ the participation of a TF P_a in binding a cis-regulatory element of G_b. To minimise the number of variables, the potential involvement of a TF combined with its effective presence/absence in a regulatory complex is represented by a unique matrix of Boolean elements $\boldsymbol{\Gamma} = \{\Gamma_{ab}\}$, with $a = 1, ..., N$ and $b = 1, ..., M$, which depend on the concentration levels $\mathbf{p}$:

$$\Gamma_{ab}(p_a(t)) = \begin{cases} 1 & \text{if } P_a \curvearrowright G_b \text{ and } p_a(t) \geq \theta_{ab} \\ 0 & \text{if } P_a \curvearrowright G_b \text{ and } p_a(t) < \theta_{ab}, \end{cases} \tag{30}$$

where $\{\theta_{ab}\}$ are concentration thresholds. Updating $\boldsymbol{\Gamma}$ at each time step, the activity of gene G_b is determined by the Boolean output of a logic function f_b, combination of the Boolean operators AND, OR, and NOT:

$$g_b = f_b(\Gamma_{1,b}(p_1), \Gamma_{2,b}(p_2), ..., \Gamma_{N,b}(p_N)) = f_b(\boldsymbol{\Gamma}_b(\mathbf{p})) \tag{31}$$

(omitting time for clarity), for example: $g_1 = (\Gamma_{1,1} \text{ OR } \Gamma_{2,1}) \text{ AND } \Gamma_{3,1}$. If f_b is a pure AND operator, then it means that all activators must be present and all repressors absent for the expression of G_b to be enhanced. If it is a pure OR operator, then a single activator suffices to enhance G_b.

Protein Synthesis. We assume here that if a gene G_b is active, i.e. $g_b = 1$, the concentration of the protein $P_{a=b}$ that it encodes simply increases with a constant rate γ_b characteristic of the gene:

$$\frac{dp_{a=b}}{dt} = \gamma_b g_b = \gamma_b f_b(\mathbf{\Gamma}_b(\mathbf{p})). \tag{32}$$

Protein Reactions. We consider elementary protein-protein reactions involving n reactants and one product, generally expressed by

$$\alpha_{a_1} P_{a_1} + \alpha_{a_2} P_{a_2} + \ldots + \alpha_{a_n} P_{a_n} \xrightarrow{k} \alpha_{a_{n+1}} P_{a_{n+1}}, \tag{33}$$

where indices $(a_1, \ldots, a_{n+1}) \in [1, N]^{n+1}$ correspond to a subset of $n+1$ proteins, k is the reaction rate coefficient, and α_{a_l} are the stoichiometric coefficients of this particular reaction. The corresponding rate equation reads

$$r^{\text{pro}}_{\{a_l\}} = k\, p_{a_1}^{x_{a_1}}\, p_{a_2}^{x_{a_2}} \ldots p_{a_n}^{x_{a_n}} = k \prod_{l=1}^{n} p_{a_l}^{x_{a_l}}, \tag{34}$$

where exponents x_{a_l} are called the "partial reaction orders" of proteins P_{a_l}, not necessarily equal to coefficients α_{a_l}. From there, the temporal evolution of all protein concentrations is given by

$$\frac{dp_{a_l}}{dt} = \begin{cases} -\alpha_{a_l} r^{\text{pro}}_{\{a_l\}} & \text{for } l \in [1, n] \\ \alpha_{a_l} r^{\text{pro}}_{\{a_l\}} & \text{for } l = n+1. \end{cases} \tag{35}$$

The special case of reactants in great excess, whose concentration is nearly constant, can be treated by setting their partial orders x_{a_l} to zero, i.e. a contribution of 1 to the rate. If a reactant is a catalyst, then a zero stoichiometric coefficient α_{a_l} also prevents it from being consumed.

Naturally, this simplified scheme has limitations. Nonetheless, when applied to many nodes of a large network of molecular interactions inside each cell, and combined with ligand secretion, extracellular diffusion, and signal transduction, it can already give rise to complex spatiotemporal dynamics in a multicellular organism or tissue.

Protein Degradation. Proteins P_a are also degraded by various processes present in the cell. We model degradation by another simple equation, based on a constant coefficient κ_a characteristic of the protein:

$$\frac{dp_a}{dt} = -\kappa_a p_a. \tag{36}$$

Protein Partial Dynamics. In sum, taking into account the effects of synthesis, reactions and degradation (but not yet ligand secretion or ligand-gated signal transduction; see below), the total variation of intracellular protein P_a is given by:

$$\frac{dp_a}{dt} = \gamma_a f_a(\mathbf{\Gamma}_a(\mathbf{p})) \pm \alpha_a r^{\text{pro}}_{\{a_l\}} - \kappa_a p_a. \tag{37}$$

Protein Programming. Exceptionally, the concentration of protein P_a can also be configured to increase by a constant rate γ_a that depends only on the spatial location of the cell and time. Clearly, this rule infringes the locality principle adopted throughout the MecaGen model, i.e. the fact that cell actions are triggered by local cues only, not global information. Yet, it constitutes a useful recourse in case of incomplete GRN specification: patterns of gene expression observed in real data can thus be "staged" and play the role of initial conditions or inputs in the simulated embryo without having to specify the cause of this expression.

This rule requires to define a time interval $T = [t_{\min}, t_{\max}]$ and a spatial domain of expression C in the shape of a rectangular cuboid between coordinates $\vec{X}_{\min} = (x_{\min}, y_{\min}, z_{\min})$ and $\vec{X}_{\max} = (x_{\max}, y_{\max}, z_{\max})$, with respect to an orthonormal basis $(\vec{e}_x, \vec{e}_y, \vec{e}_z)$ specified by the user and originating from the centre of mass of the embryo. Then, at any time step t, the spatiotemporal rate law of protein P_a in a cell i is set to

$$\frac{dp_{a,i}}{dt} = \begin{cases} \gamma_a & \text{if } t \in T \text{ and } X_i \in C \\ 0 & \text{otherwise,} \end{cases} \tag{38}$$

which replaces the synthesis equation 32 and the first term in equation 47.

The next sections introduce additional terms coming from ligand secretion and transport mechanisms, which represent biochemical and physical communication between the cell and its exterior.

Ligands. Cells in the developing embryo communicate through various means. The most common mechanisms are *secretion* out of the cell (typically by exocytosis) and *signal transduction* into the cell (via ligand-receptor interactions). Extracellular ligands are denoted here by $\{Q_c\}$, and their concentrations by $\mathbf{q} = \{q_c\}$, with $c = 1, ..., L$.

Ligand Secretion. Ligands can be externalised from the cellular domain by means of secretion. A gene output connected to the *signal secretion* rule in the GRN sends a certain quantity of its synthesized protein P_a into the space between cells, the "interstitium", creating a concentration of extracellular ligand $Q_{c=a}$ (the same molecular type as P_a, but outside the cell membrane) with a secretion rate coefficient σ_a characteristic of the ligand protein. This can be represented by the following reaction and associated rate equations:

$$P_a \xrightarrow{\sigma_a} Q_{c=a}, \text{ with } \frac{dq_{c=a}}{dt} = -\frac{dp_a}{dt} = \sigma_a p_a \quad \equiv s_a, \tag{39}$$

where s_a denotes the rate of secretion. Some extracellular ligands diffuse farther than a typical cell size, while others remain attached to the cell membrane and affect only neighbouring cells. Both scenarios are treated in the next section.

Ligand-Gated Signal Transduction. Conversely, an external signal can be converted to an intracellular messenger by *signal transduction*. Three molecular actors are involved in this process: a receptor protein R_{ac} on the membrane, the extracellular ligand Q_c binding to the receptor, and the transduced messenger protein P_a downstream of the receptor, which may or may not be the same molecular type as the ligand, i.e. $a = c$ or $a \neq c$ in our indexing of proteins. The transduction rule is active simply if its receptor is present on the cell membrane. The receptor and ligand may or may not disappear during the binding process. The following generic reaction and kinetic equations summarise these alternative scenarios:

$$\alpha_Q Q_c + \alpha_R R_{ac} \xrightarrow{\tau_c} \alpha_P P_a \;\Rightarrow\; \begin{cases} dq_c/dt = -\alpha_Q\, r_{ac}^{\text{tra}} \quad \equiv -d_c \\ d\rho_{ac}/dt = -\alpha_R\, r_{ac}^{\text{tra}} \\ dp_a/dt = \alpha_P\, r_{ac}^{\text{tra}} \end{cases}$$

$$\text{with } r_{ac}^{\text{tra}} = \tau_c\, q_c^{x_Q}\, \rho_{ac}^{x_R}, \tag{40}$$

where τ_c is the reaction rate coefficient, ρ_{ac} is the concentration of receptor, and d_c denotes the rate of ligand consumption.

This model does not discriminate, however, between receptors located on the same cell that secreted the ligand (autocrine signals) and receptors located on another cell (paracrine signals). In the particular case of a nondiffusive, transmembrane ligand that remains attached to its source cell's surface and binds to a receptor on a neighbour cell across the gap (such as the Delta-Notch mechanism in Fig. 4), the reaction rate r_{ac}^{tra} above is replaced with a sum of contributions over the neighbours' ligands:

$$\alpha_Q Q_{c,j} + \alpha_R R_{ac,i} \xrightarrow{\tau_c} \alpha_P P_{a,i}$$
$$\text{with} \quad r_{ac,i}^{\text{tra}} = \tau_c \sum_{j \in \mathcal{N}_i} (q_{c,j} \frac{A_{ij}}{A_j})^{x_Q} (\rho_{ac,i} \frac{A_{ij}}{A_i})^{x_R}, \tag{41}$$

where A_{ij} is the surface of contact between the cells, and A_i and A_j are their total surface areas.

Ligand Degradation. Similarly to intracellular proteins, extracellular ligands Q_c can also be degraded by various unmodelled molecules present in the interstitium. We summarise this process through a simple term regulated by a constant coefficient χ_c characteristic of the ligand:

$$\frac{dq_c}{dt} = -\chi_c q_c. \tag{42}$$

Ligand Diffusion. The infrastructure of chemical signalling in the model corresponds to the same graph of topological neighbourhoods as the one used by force mechanics. External ligands diffuse in the interstitial gaps between cells, delimited by their membranes, therefore the links connecting the centres of the cells are the "dual" representation of this space. Such network also offers a spatial representation of the embryo that is robust with respect to the deformation of the multicellular assembly.

The macroscopic dynamic describing the diffusion of molecules is based on Fick's law. It states that ligands move from regions of high concentration to regions of low concentration with an amplitude proportional to the spatial gradient of the concentration. Generally, the flux $\vec{J_c}$ measuring the quantity of extracellular ligand Q_c passing through a small section of space during a small time interval is given by:

$$\vec{J_c} = -D_c \vec{\nabla} q_c, \tag{43}$$

where D_c is the diffusion coefficient of the ligand and $q_c = q_c(x,y,z)$ its concentration field. In our network of cells, Q_c flows "on the edges" between each node i and its neighbours j (in one direction or the other). Denoting by $q_{c,i}$ the concentration of Q_c localised near the surface of cell i, and by $\vec{J}_{c,ij}$ the flux of Q_c between i and j, we can write the discrete approximation:

$$\vec{J}_{c,ij} = -D_c \frac{q_{c,j} - q_{c,i}}{r_{ij}} \vec{u}_{ij}, \tag{44}$$

where, as before, r_{ij} is the distance between i and j, and $\vec{u}_{ij}$ the unit vector from i to j (Supplementary Fig. 10). Note that this vector is invariant by reversal of direction: $\vec{J}_{c,ij} = \vec{J}_{c,ji}$, consistent with the hypothesis of a unique concentration field of Q_c between i and j, irrespective of the viewpoint.

Ligand Total Dynamics. The temporal evolution of the concentration is determined by the *continuity equation*, which is a local form of conservation law. The "divergence theorem" gives the integral form of the continuity equation, applied on the volume of the cell. Its continuous expression reads

$$\frac{\partial q_c}{\partial t} + \iint_O \vec{J_c} \cdot \vec{dA} = s_c - d_c - \chi_c q_c, \tag{45}$$

where $\vec{dA}$ is the normal vector of the closed surface of the cell, s_c is the "source" term corresponding to the rate of extracellular Q_c ligand produced by secretion (if any), and $-d_c - \chi_c q_c$ is the "sink" term corresponding to the rate of ligand disappearing by transduction and degradation (if any).

Finally, reintroducing the cell's index i, the discrete approximation of this closed surface integral involves the topological neighbourhoods $\mathcal{N}_i$ and contact areas A_{ij} as follows:

$$\begin{aligned}\frac{\partial q_{c,i}}{\partial t} &= s_{c,i} - d_{c,i} - \chi_c q_{c,i} - \sum_{j \in \mathcal{N}_i} \|\vec{J}_{c,ij}\| A_{ij} \\ &= \sigma_c p_{c,i} - \alpha_Q \sum_{a:\rho_{ac}>0} r_{ac}^{\text{tra}} - \chi_c q_{c,i} - D_c \sum_{j \in \mathcal{N}_i} \frac{A_{ij}}{r_{ij}} (q_{c,j} - q_{c,i}). \end{aligned} \tag{46}$$

where some of the terms can be omitted by setting their respective coefficient to 0 if the rule does not apply.

Protein Total Dynamics. Finally, coming back to the intracellular protein dynamics, we can combine equation 37 with ligand secretion (equation 39) and standard ligand-gated transduction (equation 40) to obtain the complete equation of intracellular protein variation (where terms are also independent and optional):

$$\frac{dp_a}{dt} = \gamma_a f_a(\mathbf{\Gamma}_a(\mathbf{p})) \pm \alpha_a r_{\{a_l\}}^{\text{pro}} - (\kappa_a + \sigma_a) p_a + \alpha_P \sum_{c:\rho_{ac}>0} r_{ac}^{\text{tra}}. \tag{47}$$

Supplementary Note 4 | MecaGen: model of mechanic-genetic coupling

The last sections below describe how the mechanical parameters and archetype are controlled by the genetic and molecular dynamics, and vice-versa, through the CBO.

Control of Attraction by Surface Densities of Adhesion Molecules. Passive relaxation forces due to cell adhesion are relatively straightforward to link to the output of molecular signalling and genetic regulation. The intensity of adhesion between two cells depends on the surface densities of their adhesion molecules, which is assumed uniformly distributed on the cell membranes. At the interface between i and j, we define the *surface concentration* $c_{a,ij}$ (resp. $c_{a,ji}$) of protein P_a on the membrane of i (resp. j) as a fraction of the intracellular protein concentrations $p_{a,i}$ and $p_{a,j}$:

$$\begin{cases} c_{a,ij} &= p_{a,i} A_{ij}/A_i \\ c_{a,ji} &= p_{a,j} A_{ij}/A_j, \end{cases} \tag{48}$$

where A_{ij} is the surface of contact between the cells, and A_i and A_j are their total surface areas. Then, following the example of Zhang et al.,[26] we consider three possible binding models relating $c_{a,ij}$ to the attraction-repulsion force $\vec{F}_{ij}^{\mathrm{P,ar}}$ via protein-specific adhesion coefficents $w_{a,ij}^{\mathrm{adh}}$:

- the *cis-dimer* model (CDM), applying to adhesion molecules that bind at the cell-cell interface after having formed cis-dimers on their respective face:[27]

$$w_{a,ij}^{\mathrm{adh}} = k_a^{\mathrm{cdm}}\, c_{a,ij}^2\, c_{a,ji}^2, \tag{49}$$

- the *trans-homophilic-bond* model (THBM), applying to adhesion molecules that bind individually at the cell-cell interface:[28]

$$w_{a,ij}^{\mathrm{adh}} = k_a^{\mathrm{thbm}}\, c_{a,ij}\, c_{a,ji}, \tag{50}$$

- the *saturation* model (SM), applying to adhesion molecules that form large clusters and saturate:

$$w_{a,ij}^{\mathrm{adh}} = k_a^{\mathrm{sm}} \min\{c_{a,ij},\, c_{a,ji}\}. \tag{51}$$

where factors k_a are characteristic of molecular species P_a and its associated mode of binding in each model. Note that the resulting molecular adhesion coefficient is symmetrical in all cases: $w_{a,ij}^{\mathrm{adh}} = w_{a,ji}^{\mathrm{adh}}$. With multiple types of adhesion molecules P_a coexisting between two adjacent cells, we assume that each of them is involved independently in "homotypic" adhesion, i.e. binds only its own type. Therefore, the total adhesion coefficient between i and j is a simple linear sum of these contributions: $w_{ij}^{\mathrm{adh}} = \sum_a w_{a,ij}^{\mathrm{adh}}$, where index a covers a subset of $[1,N]$ representing the family of adhesion proteins. This custom value replaces w_{adh} in equation (14), the expression of the attraction-repulsion force.

Control of Archetype and Shape by Gene Regulation and Neighbours. A cell's internal protein state determines which archetype it will adopt. This is achieved by connecting archetype "nodes", mesenchymal and/or epithelial, to the GRN in output (Fig. 1b,4a) via the same type of Boolean functions used for gene regulatory sites, denoted here by $f_{\mathcal{M}}$ and $f_{\mathcal{E}}$ (equations 30,31). Ideally, the topology of the GRN specified by the modeller should be such that only one of several concurrent archetypes can be selected, for example by introducing feedback inhibition or attractor states. This follows the frequent observation that certain genes are mutually exclusive and cannot be simultaneously active.[29,30] If none of these nodes is activated, the default idle archetype $\mathcal{I}$ is selected for the cell.

Additionally, the epithelial archetype also depends on the requirement to distinguish between a functionally active polarised cell ($\mathcal{E}_\pm$) and an unpolarised cell ($\mathcal{E}_0$). To be epithelial, a cell must be surrounded by at least two other epithelial neighbour cells and form a triangle $\widehat{X_{a_k} X_i X_{a_{k+1}}}$ whose angle is less than π. This condition conveys the idea that lateral reinforcement is required between neighbour epithelial cells to define a proper epithelium. If this condition is not fullfilled, the unpolarised cell behaves like an $\mathcal{I}$ cell. If a cell i becomes epithelial, however, its shape switches from spherical (single radius R_i) to ellipsoidal (two radii $R_i^{\parallel}$ and $R_i^{\perp}$; equations 1,2).

Control of Polarisation by Gene Regulation, Ligands and Neighbours. Every type of "active" intercalating behaviour displayed by cell i, whether mesenchymal protrusion, or epithelial junction remodelling, requires a polarisation axis. In real cells, polarisation correlates with an asymmetry of intracellular molecular concentrations. In our model, since we made the choice of one particle per cell, there is no explicit spatial distribution of intracellular material. Instead, we represent this chemical asymmetry by vectors $\vec{U}_i$ passing through the centres of the cells (the special case of the AB axis $\vec{U}_i^{\text{ab}}$ in epithelial cells was treated geometrically in equation 9).

More precisely, a cell can be polarised by several different mechanisms throughout development, corresponding to several "potential" polarisation axes, each of them determined by one of four modes and various protein types: (a) a local gradient-based, or "chemotactic" mode, (b) a propagation mode by contact between cells, (c) a protrusion-induced mode, and (d) a default, "blebbing" mode if there is no input into the cell to trigger one of the above three mechanisms (until another mode takes over). The selection of one of these axes by the GRN dynamics is explained below.

- **(a) Chemotactic mode:** According to a classical view of polarisation, a cell is able to detect local asymmetries of extracellular ligand concentration in its vicinity. The resulting axis is calculated here by a weighted average of the neighbourhood edges $\vec{u}_{ij}$, using the abstract graph of neighbourhood relationships $\mathcal{N}_i$ and the extracellular ligand quantities q_a:

$$\vec{U}_{a,i}^{\text{chem}} = \sum_{j \in \mathcal{N}_i} (q_{a,j} - q_{a,i})^m \vec{u}_{ij}, \tag{52}$$

  where $q_{a,i}$ is the local concentration of Q_a near i, and m is an integer exponent controlling the sensitivity of detection of the ligand differentials (m must be odd to conserve vector directions, typically $m = 3$).

- **(b) Propagation mode:** A cell can also sense its neighbours' polarisation (via chemical surfacic cues reflecting their internal spatial assymmetries) and will tend to "align" its own axis with theirs via a weighted average:

$$\vec{U}_{a,i}^{\text{prop}} = \sum_{j \in \mathcal{N}_i} q'_{a,j} \vec{U}_{a,j}^{\text{prop}} \quad \text{with} \quad \begin{cases} q'_{a,j} = q_{a,j} & \text{if } q_{a,j} \geq \theta_a \\ q'_{a,j} = 0 & \text{if } q_{a,j} < \theta_a, \end{cases} \tag{53}$$

  where θ_a is a sensitivity threshold defining a minimal ligand quantity under which the neighbouring cell j cannot be detected, thus does not exert an influence on i. This recursive relationship creates a "wave" of axis alignment, which is initialised by setting $\vec{U}_{a,i}^{\text{prop}} = \vec{U}_{a,i}^{\text{chem}}$ in some cells. Typically, this would happen on the border of a morphogenetic field, such as the epithelial populations of the second case study (Main Article, "Compartmentalisation and epithelialisation").

- **(c) Protrusion-induced mode:** Recent studies have unveiled a novel mechanism for the propagation of polarisation axis based on mechanical interactions.[31,32] Weber et al.[31] showed that a mechanical traction exerted at one end of a *Xenopus* cell triggered a protrusion in the opposite direction through

a reorganisation of internal keratin filaments. In MecaGen, this mechanism is idealised by orienting the potential polarisation axis of a cell i in the direction opposite to the average force exerted by the neighbouring cells j protruding on and around it:

$$\vec{U}_i^{\text{prin}} = -\sum_{j\in\mathcal{N}_i} \vec{F}_{ij}^{\text{A,ext}}, \tag{54}$$

where $\vec{F}_{ij}^{\text{A,ext}}$ is the "extrinsic active" force exerted by j over i.

- **(d) Blebbing mode:** When a differentiated cell does not receive spatial cues about how to orient itself, its polarisation axis $\vec{U}_i^{\text{bleb}}$ is randomly and (quasi) uniformly set anywhere on the unit sphere in the same way as equation (25) at regular time intervals, typically every 5 simulated minutes.

The internal protein state, controlled by the GRN dynamics, determines which polarisation mode among the four described above will be activated next and involved in the cell's behavioural activity. Similar to the archetypes, these modes are connected to the GRN in output (Fig. 1b,4a) via Boolean functions f_{chem}, f_{prop}, f_{prin}, and f_{bleb} (equations 30,31). It means that if a given group of associated regulatory proteins is present in sufficient quantities, they will trigger the corresponding vector $\vec{U}_i$ to be the next effective axis. Again, the modeller must ensure that the molecular states induced by the GRN topology cannot activate several modes at the same time. To help prevent any ambivalence in the polarisation state, the user can also assign "priority" rankings to polarisation modes, except for $\vec{U}_i^{\text{prin}}$, which is always dominated by another active mode, and $\vec{U}_i^{\text{bleb}}$, which is selected only if no other mode is active.

After one of these modes has been selected by the GRN dynamics, the new axis $\vec{U}_i^{\text{pm}}$ is renormalised and combined with the current axis $\vec{U}_i$ via a "memory" coefficient $\omega \geq 0$, then the result is renormalised again:

$$\vec{U}_i \leftarrow \omega \vec{U}_i + \frac{\vec{U}_i^{\text{pm}}}{\|\vec{U}_i^{\text{pm}}\|}, \quad \text{followed by} \quad \vec{U}_i \leftarrow \frac{\vec{U}_i}{\|\vec{U}_i\|}. \tag{55}$$

In the case of epithelial cells, however, the polarisation axis must be oriented either along the AB axis or on the plane orthogonal to the AB axis. This is specified for each potential axis potentially involved in epithelial polarisation by a binary variable ψ. Therefore, if $i \in \mathcal{E}$, a special update dynamics is applied:

$$\begin{cases} \vec{U}_i \leftarrow \vec{U}_i^{\text{ab}} & \text{if } \psi = 1 \\ \vec{U}_i \leftarrow \vec{U}_i^{\text{pm}} - (\vec{U}_i^{\text{pm}} \cdot \vec{U}_i^{\text{ab}})\vec{U}_i^{\text{ab}} & \text{if } \psi = 0, \end{cases} \quad \text{followed by} \quad \vec{U}_i \leftarrow \frac{\vec{U}_i}{\|\vec{U}_i\|}. \tag{56}$$

Control of Protrusion Behaviour by Gene Regulation. Given the polarisation axis $\vec{U}_i$, another set of rules creates a coupling from the GRN state to the cell's biomechanical behaviour, in particular its protrusion activity, via the active behavioural forces. Like the archetype and polarisation axis, these rules are regulated by a genetic Boolean function f_{prot}, i.e. the absence or presence of a given set of intracellular regulatory proteins (equations 30,31). In the positive case, if a cell i actually adopts a protrusive behaviour on a neighbouring cell j, then the coefficient φ_{prot} of its corresponding active force (used in equation 21) is set to a value proportional to the adhesion coefficient, and possibly local to the ij contact:

$$\varphi_{ij}^{\text{prot}} = \phi w_{a,ij}^{\text{adh}} \quad \text{or} \quad \varphi_{\text{prot}} = \phi w_{\text{adh}}, \tag{57}$$

depending on whether there is also a control of attraction forces via a specific adhesion molecule P_a (equations 49-51) or not, and where ϕ is the coupling coefficient. In any case, this formulation takes into account the role of focal molecular adhesion complexes in the protrusion efficiency. Since the orientation and direction of protrusion is determined by the cell's polarisation axis $\vec{U}_i$ (and its orthogonal complement $\vec{U}_i^{\perp j}$ passing through j), the GRN must be designed to activate the desired polarisation mode in concert with the protrusion mode.

Control of Gene Regulation by Mechanotransduction. Recent studies have shown that certain genes can also be upregulated by the mechanical forces that are stretching or pressing on the cell.[33] This principle is also part of the MecaGen platform, as it contributes to the Meca→Gen coupling direction. Practically, it means adding a new rule to the molecular and genetic side, which becomes activated if the sum of the force magnitudes exerted on the cell is greater than a mechanotransduction threshold, denoted by F_a^{mt}. This rule simply increases the concentration variation rate of a target protein P_a by a characteristic constant ξ_a, whichever value this rate had reached after collecting other influences from genes, proteins, secretion, or transduction:

$$\sum_{j \in \mathcal{N}_i} \|\vec{F}_{ij}\| \geq F_a^{\mathrm{mt}} \quad \Rightarrow \quad \frac{dp_a}{dt} \leftarrow \frac{dp_a}{dt} + \xi_a. \tag{58}$$

Supplementary Note 5 | Implementation and computation

Computational speed and scalability. The whole MecaGen model was designed from the start to achieve fast computation times, keeping in mind the possibility of parameter space exploration on computing clusters (Fig. 6d, Supplementary Note 3). On today's laptops, simple MecaGen simulations involving a few 100 cells typically take a few seconds, while more complex experiments involving a few 1000 cells take a few minutes. The measurements below were made for simulations running on a single core, with graphical user interface (GUI) and Cuda/VBO rendering, on two differents machines (shorter computation times can be obtained with GUI turned off).

	Number of cells (start→end)	Number of time steps	Computation time in total		Computation time per step	
			CPU1*	CPU2**	CPU1*	CPU2**
Patterning study (Fig. 3)	880→1125	1000	4.4 s	3.8 s	4.4 ms	3.8 ms
Compartment study (Fig. 4)	1683	800	14.9 s	13.5 s	18 ms	16 ms
Epiboly study (Fig. 5,6)	1548→3095	4000	173.8 s	146.3 s	43 ms	36 ms

*Intel Core i7-4712HQ @ 2.30GHz, **Intel Core i5-3570K @ 3.40GHz

Choice of MecaGen parameter values. In the first two case studies presented here (Fig. 3,4), which were illustrative examples without specific biological questions, parameters received fixed values chosen by hand (Supplementary Tables 2-4). In the third case study (Fig. 5), a more systematic search and optimisation was conducted across two parameters related to the adhesion forces (Fig. 6d). The objective here was to estimate parameters able to qualitatively reproduce typical macroscopic behaviours. Additionally, since some parameters offset each other, orders of magnitude could have been chosen differently. For example, the damping coefficient λ (equation 3) is counterbalanced by the force coefficients w_{adh}, w_{rep} and k_{rig} (equations 14,17). On the genetic side, the synthesis and degradation rates γ, κ and χ (equations 32,36,42) were set first, then the molecular interaction parameters, such as concentration thresholds θ_{ab}, regulation functions f_b (equations 30,31), or reaction rates k (equation 34), were adjusted accordingly. All values were reevaluated a few times until the expected outcome was obtained.

Exploration of MecaGen parameter space. The third case study shows that the MecaGen framework was designed to be compatible with massive model exploration and parametric search software, such as OpenMOLE[34] (Open MOdeL Experiment, http://openmole.org). Within selected regions of parameter space, this allows exhaustive reverse-engineering strategies guided by an "order parameter" evaluating the adequacy of the virtual embryo with respect to the observed specimen, and obtaining a biologically meaningful interpretation of the simulation outcome. This comparison can be based on macroscopic geometric measurements, as in the epiboly study (Fig. 6a,b), or degrees of overlap between cell lineages or vector fields at a miscroscopic scale. All 3D+time cell positions and molecular states can be exported from the simulations and confronted to live embryo data extraction and reconstruction. More refined metaheuristic methods such as evolutionary algorithms, with possible human interaction, for example using the EASEA platform[35] (EAsy Specification of Evolutionary Algorithms, http://easea.unistra.fr), can also be employed. Mutation operators are applied to the GRN and mechanical parameters, then developed individuals can be selected on the basis of their "fitness" or, on the contrary, specific pathologies under investigation.

Supplementary Note 6 | MecaGen source code and installation. *MecaGen runs on Linux only*. Mac OS and Windows systems are not supported. Installation and execution were successfully tested on Ubuntu and Arch Linux, and are expected to work on other Linux distributions, too. The complete MecaGen package is publicly released under the GNU General Public License v3.0 after publication of the article via our project's website http://www.mecagen.org. We also provide two tutorial videos demonstrating the instructions: http://youtu.be/5zcLAL-caDQ (Thrust, default mode) and http://youtu.be/d79v7MDPIBw (CUDA compilation, "Zebrafish" mode).

Supplementary References

1. Thompson, D. W. *On growth and form* (Cambridge Univ. Press, 1942).
2. Turing, A. M. The chemical basis of morphogenesis. *Philos. T. Roy. Soc. B: Biological Sciences* **237**, 37–72 (1952).
3. Wolpert, L. Positional information and the spatial pattern of cellular differentiation. *J. of Theor. Biol.* **25**, 1–47 (1969).
4. Gierer, A. & Meinhardt, H. A theory of biological pattern formation. *Kybernetik* **12**, 30–39 (1972).
5. Weliky, M. & Oster, G. The mechanical basis of cell rearrangement. *Development* **109**, 373–386 (1990).
6. Weliky, M., Minsuk, S., Keller, R. & Oster, G. Notochord morphogenesis in xenopus laevis: simulation of cell behavior underlying tissue convergence and extension. *Development* **113**, 1231–1244 (1991).
7. Mjolsness, E., Sharp, D. H. & Reinitz, J. A connectionist model of development. *J. Theor. Biol.* **152**, 429–453 (1991).
8. Glazier, J. A. & Graner, F. Simulation of the differential adhesion driven rearrangement of biological cells. *Phys. Rev. E* **47**, 2128 (1993).
9. Shapiro, B. E. & Mjolsness, E. D. Developmental simulations with cellerator. In *Proc. ICSB*, vol. 435 (2001).
10. Marée, A. F. M. & Hogeweg, P. How amoeboids self-organize into a fruiting body: multicellular coordination in dictyostelium discoideum. *PNAS* **98**, 3879–3883 (2001).
11. Meir, E., Munro, E. M., Odell, G. M. & Von Dassow, G. Ingeneue: a versatile tool for reconstituting genetic networks, with examples from the segment polarity network. *J. Exp. Zool.* **294**, 216–251 (2002).
12. Dallon, J. C. & Othmer, H. G. How cellular movement determines the collective force generated by the dictyostelium discoideum slug. *J. Theor. Biol.* **231**, 203–222 (2004).
13. Schaller, G. & Meyer-Hermann, M. Multicellular tumor spheroid in an off-lattice voronoi-delaunay cell model. *Phys. Rev. E* **71**, 051910 (2005).
14. Robertson, S. H. *et al.* Multiscale computational analysis of xenopus laevis morphogenesis reveals key insights of systems-level behavior. *BMC Sys. Biol.* **1**, 46 (2007).
15. Käfer, J., Hayashi, T., Marée, A. F. M., Carthew, R. W. & Graner, F. Cell adhesion and cortex contractility determine cell patterning in the drosophilaretina. *PNAS* **104**, 18549–18554 (2007).
16. Krieg, M. *et al.* Tensile forces govern germ-layer organization in zebrafish. *Nat. Cell Biol.* **10**, 429–436 (2008).
17. Honda, H., Motosugi, N., Nagai, T., Tanemura, M. & Hiiragi, T. Computer simulation of emerging asymmetry in the mouse blastocyst. *Development* **135**, 1407–1414 (2008).
18. Rejniak, K. A. & Anderson, A. R. A. A computational study of the development of epithelial acini: I. sufficient conditions for the formation of a hollow structure. *B. Math. Biol.* **70**, 677–712 (2008).
19. Hoehme, S. & Drasdo, D. A cell-based simulation software for multi-cellular systems. *Bioinformatics* **26**, 2641–2642 (2010).
20. Sandersius, S. A., Chuai, M., Weijer, C. J. & Newman, T. J. Correlating cell behavior with tissue topology in embryonic epithelia. *PloS ONE* **6**, e18081 (2011).
21. Hester, S. D., Belmonte, J. M., Gens, J. S., Clendenon, S. G. & Glazier, J. A. A multi-cell, multi-scale model of vertebrate segmentation and somite formation. *PLoS Comp. Biol.* **7**, e1002155 (2011).
22. Sandersius, S. A., Chuai, M., Weijer, C. J. & Newman, T. J. A "chemotactic dipole" mechanism for large-scale vortex motion during primitive streak formation in the chick embryo. *Phys. Biol.* **8**, 045008 (2011).
23. Merks, R. M., Guravage, M., Inzé, D. & Beemster, G. T. Virtualleaf: an open-source framework for cell-based modeling of plant tissue growth and development. *Plant Physiol.* **155**, 656–666 (2011).
24. Shapiro, B. E., Meyerowitz, E. M. & Mjolsness, E. Using cellzilla for plant growth simulations at the cellular level. *Front. Plant Sci.* **4** (2013).
25. Tanaka, S., Sichau, D. & Iber, D. Lbibcell: a cell-based simulation environment for morphogenetic problems. *Bioinformatics* btv147 (2015).
26. Zhang, Y., Thomas, G. L., Swat, M., Shirinifard, A. & Glazier, J. A. Computer Simulations of Cell Sorting Due to Differential Adhesion. *PLoS ONE* **6**, e24999 (2011).
27. Pertz, O. *et al.* A new crystal structure, ca2+ dependence and mutational analysis reveal molecular details of e-cadherin homoassociation. *The EMBO Journal* **18**, 1738–1747 (1999).
28. Boggon, T. J. *et al.* C cadherin ectodomain structure and implications for cell adhesion mechanisms. *Science* **296**, 1308–1313 (2002).
29. Jaeger, J. *et al.* Dynamic control of positional information in the early Drosophila embryo. *Nature* **430**, 368–371

(2004).
30. Balaskas, N. *et al.* Gene regulatory logic for reading the Sonic Hedgehog signaling gradient in the vertebrate neural tube. *Cell* **148**, 273–84 (2012).
31. Weber, G. F., Bjerke, M. A. & Desimone, D. W. A mechanoresponsive cadherin-keratin complex directs polarized protrusive behavior and collective cell migration. *Developmental Cell* **22**, 104–115 (2012).
32. Dumortier, J. G., Martin, S., Meyer, D., Rosa, F. M. & David, N. B. Collective mesendoderm migration relies on an intrinsic directionality signal transmitted through cell contacts. *Proceedings of the National Academy of Sciences* **109**, 16945–16950 (2012).
33. Desprat, N., Supatto, W., Pouille, P.-A., Beaurepaire, E. & Farge, E. Tissue deformation modulates twist expression to determine anterior midgut differentiation in Drosophila embryos. *Developmental Cell* **15**, 470–477 (2008).
34. Reuillon, R., Leclaire, M. & Rey-Coyrehourcq, S. Openmole, a workflow engine specifically tailored for the distributed exploration of simulation models. *Future Gener. Comp. Sy.* **29**, 1981–1990 (2013).
35. Maitre, O., Baumes, L. A., Lachiche, N., Corma, A. & Collet, P. Coarse grain parallelization of evolutionary algorithms on GPGPU cards with EASEA. In *Proc. GECCO*, 1403–1410 (ACM, 2009).
